# Genetic loci associated with coronary artery disease harbor evidence of selection and antagonistic pleiotropy

**DOI:** 10.1371/journal.pgen.1006328

**Published:** 2017-06-22

**Authors:** Sean G. Byars, Qin Qin Huang, Lesley-Ann Gray, Andrew Bakshi, Samuli Ripatti, Gad Abraham, Stephen C. Stearns, Michael Inouye

**Affiliations:** 1 Centre for Systems Genomics, School of BioSciences, The University of Melbourne, Parkville, Victoria, Australia; 2 Department of Pathology, The University of Melbourne, Parkville, Victoria, Australia; 3 Baker Heart and Diabetes Institute, Melbourne, Victoria, Australia; 4 Institute of Molecular Medicine Finland, University of Helsinki, Helsinki, Finland; 5 Department of Public Health, University of Helsinki, Helsinki, Finland; 6 Wellcome Trust Sanger Institute, Wellcome Genome Campus, Hinxton, Cambridge, United Kingdom; 7 Department of Ecology and Evolutionary Biology, Yale University, New Haven, CT, United States of America; Geisinger Health System, UNITED STATES

## Abstract

Traditional genome-wide scans for positive selection have mainly uncovered selective sweeps associated with monogenic traits. While selection on quantitative traits is much more common, very few signals have been detected because of their polygenic nature. We searched for positive selection signals underlying coronary artery disease (CAD) in worldwide populations, using novel approaches to quantify relationships between polygenic selection signals and CAD genetic risk. We identified new candidate adaptive loci that appear to have been directly modified by disease pressures given their significant associations with CAD genetic risk. These candidates were all uniquely and consistently associated with many different male and female reproductive traits suggesting selection may have also targeted these because of their direct effects on fitness. We found that CAD loci are significantly enriched for lifetime reproductive success relative to the rest of the human genome, with evidence that the relationship between CAD and lifetime reproductive success is antagonistic. This supports the presence of antagonistic-pleiotropic tradeoffs on CAD loci and provides a novel explanation for the maintenance and high prevalence of CAD in modern humans. Lastly, we found that positive selection more often targeted CAD gene regulatory variants using HapMap3 lymphoblastoid cell lines, which further highlights the unique biological significance of candidate adaptive loci underlying CAD. Our study provides a novel approach for detecting selection on polygenic traits and evidence that modern human genomes have evolved in response to CAD-induced selection pressures and other early-life traits sharing pleiotropic links with CAD.

## Author summary

How genetic variation contributes to disease is complex, especially for those such as coronary artery disease (CAD) that develop over the lifetime of individuals. One of the fundamental questions about CAD––whose progression begins in young adults with arterial plaque accumulation leading to life-threatening outcomes later in life––is why natural selection has not removed or reduced this costly disease. It is the leading cause of death worldwide and has been present in human populations for thousands of years, implying considerable pressures that natural selection should have operated on. Our study provides new evidence that genes underlying CAD have recently been modified by natural selection and that these same genes uniquely and extensively contribute to human reproduction, which suggests that natural selection may have maintained genetic variation contributing to CAD because of its beneficial effects on fitness. This study provides novel evidence that CAD has been maintained in modern humans as a by-product of the fitness advantages those genes provide early in human lifecycles.

## Introduction

It is well established that modern human traits are a product of past evolutionary forces that have shaped heritable variation, but we are far from a good understanding of whether recent natural selection has acted on these and how this process has left its imprint across the genome. While many genome-wide multi-population scans have searched for signatures of positive selection [1–9], these studies have detected relatively few adaptive candidates for common traits or diseases [10–12]. This suggests that classic ‘selective sweeps’ have been relatively rare in recent human history [13–16] and that current tools may not be appropriate for detecting and validating smaller shifts in adaptive variation, thus limiting our understanding of how natural selection acts on common diseases and traits [12]. Research in this area is also important as the combination of positive selection and significant GWAS signals at the same locus supports the existence of functional variation for disease. Here, we aimed to comprehensively identify selection signals for coronary artery disease (CAD) loci with methods designed to detect recent signals of positive selection. We compared selection signals in 12 worldwide populations (HapMap3) with CAD genetic risk (CARDIoGRAMplusC4D) to help understand how selection acts on disease variation at the genetic level and prioritize genes most likely modified in relation to CAD. We examined the association between selection signals and gene expression to further test whether adaptive candidates are functionally important for CAD in terms of gene regulation. Lastly, we tested if CAD genes are associated with reproductive fitness to try to understand why this common disease persists in modern humans.

Classic population genetics theory describes positive selection with the selective-sweep (or hard-sweep) model, in which a strongly advantageous mutation increases rapidly in frequency (often to fixation) resulting in reduced heterozygosity of nearby neutral polymorphisms due to genetic hitch-hiking [17, 18] and a longer haplotype with higher frequency. Many methods have been developed to detect these signatures [19, 20], including traditional tests that detect differentiation in allele frequencies among populations (i.e. Wright’s fixation index, Fst [21]) and more recently developed within population tests for extended haplotype homozygosity (i.e. integrated haplotype score, iHS [9]). Some of the most convincing examples of human adaptive evolution have been uncovered for traits influenced by single loci with large effects. For example, the lactase persistence (*LCT*) and Duffy-null (*DARC*) mutations affecting expression of key proteins in milk digestion [10] and malarial resistance [22] both display hallmarks of selective sweeps. Other loci that are not clearly monogenic but also show selective sweeps are associated with high-altitude tolerance (*EPAS1* [23]) and skin pigmentation (*SLC24A5* and *KITLG* [24]). These studies show that rapid selective sweeps mainly occurred for new mutations with large effects on phenotypes.

Motivated by these initial successes and the increasing availability of global population data genotyped on higher resolution arrays (i.e. HapMap Project, 1000 Genomes Project), many recent genome-wide scans for candidate adaptive loci have recently been performed [11]. These suggest that selection may have operated on a variety of biological processes [10] in ways that differ among populations (i.e. local adaptation) [25], been prevalent in genetic variation linked to metabolic processes [26], and may often target intergenic regions and gene regulatory variants rather than protein-coding regions [12]. Often only the larger signals underlying monogenic (or near-monogenic) traits are typically considered for follow-up because of losses in the statistical power needed to quantify significance for smaller candidate adaptive signals after correcting for genome-wide multiple testing [20]. The adaptive status of many smaller candidate signals also remains uncertain due to inconsistencies in results between studies that utilized the same data [14], and it is inherently more difficult to functionally validate candidate adaptive signals underlying complex polygenic traits compared to monogenic traits where only one or a few variants may have been under selection due to their influence on fitness [27, 28].

In contrast to population genetics, research in quantitative genetics has shown that rapid adaptation can often occur on complex traits that are highly polygenic [29, 30]. Under the ‘infinitesimal (polygenic) model’, such traits are likely to respond quickly to changing selective pressures through smaller allele frequency shifts in many polymorphisms already present in the population [13, 31]. Selection on such variation is generally less likely to push it towards fixation due to genetic correlations, thus producing smaller changes in surrounding heterozygosity over time that are harder to detect with most current population genetic methods [14, 28, 32]. Note that polygenic and classic sweep models are not mutually exclusive [13, 33], for alleles with small- and large-effects may both underlie a polygenic trait, which suggests that there will be some variation in the degree to which candidate alleles are modified after selective events. Because most common diseases are highly polygenic, we need to improve how we detect and classify the adaptive signatures underlying these traits.

Recent studies investigating genomic selection on polygenic traits have taken two approaches. The first scans for significant selection signals for a subset of large effect SNPs that have previously been identified as genome-wide significant. For example, Ding and Kullo [34] found significant population differentiation (Fst) for 8 of 158 index SNPs underlying 36 cardiovascular disease phenotypes, and Raj et al. [35] observed elevated positive selection scores (Fst, iHS) for 37 of 416 index susceptibility SNPs underlying 10 inflammatory-diseases. The second approach tests if aggregated shifts in genome-wide significant allele frequencies are associated with phenotypic differences by population, latitudinal, or environmental gradients, which might indicate local adaptation. For example, Castro and Feldman [36] used 1300 index SNPs underlying many polygenic traits and found elevated adaptive signals (Fst and iHS) above background variation, and Turchin et al. [37] demonstrated moderately higher frequency of 139 height-increasing alleles in a Northern (taller) compared to Southern (shorter) European populations. These approaches all assume that genome-wide significant variants are the most probable selection targets, but many if not most such variants are tags for the causal variants, which may be at lower frequencies. This suggests an approach more sensitive for detecting subtle signals of polygenic selection is needed.

We chose CAD as a model for examining polygenic selection signals for complex disease because it has (and continues to) impose considerable disease burden (and possible selection pressure) in humans [38], its underlying genetic architecture has been extensively studied [39, 40] and many of its risk factors (cholesterol, blood pressure) have been under recent natural selection [41] related to potential pleiotropic effects or tradeoffs with CAD. Antagonistic pleiotropy describes gene effects on multiple linked traits where selection on one may cause negative fitness effects (i.e. reproduction, survival, and disease) in the other due to their antagonistic genetic association [42]. Two common misconceptions are that CAD only occurs in older people and is a disease that has mainly afflicted modern humans. If either were true, selection might not have had either the opportunity or sufficient time to affect genetic variation associated with CAD. However, CAD begins to manifest during reproductive ages [43, 44] and disease origins can be detected even in adolescence through degree of atherosclerosis [44, 45] and myocardial infarction events [46]. CAD is also a product of many heritable risk factors (cholesterol, weight, blood pressure) whose variation is expressed during the reproductive period, when CAD could drive selection directly or indirectly. Furthermore, CAD has impacted human populations since at least the ancient Middle Kingdom period, with atherosclerosis detectable in Egyptian mummies [47]. This suggests that there has been enough time for evolutionary responses to CAD to have occurred, genomic signatures from which may be detectable in modern humans.

By combining several 1000 Genomes-imputed datasets including HapMap3 and Finnish SNP data, a large genetic meta-analysis of CAD, HapMap3 gene expression data and lifetime fitness data from the Framingham Heart Study, we sought to address the reason(s) why CAD exists in humans by answering the following questions: 1) Has selection recently operated on CAD loci? 2) How do selection signals underlying CAD loci vary among populations and are they enriched for gene regulatory effects? 3) Do candidate adaptive signatures overlap directly with CAD genetic risk and is this useful for highlighting disease-linked selection signals? 4) Do CAD-linked selection signals display functional effects and evidence of antagonistic pleiotropy, in that they are also linked to biological processes or traits influencing reproduction?

## Results

To test for selection signals for variants directly linked with CAD, we utilized SNP summary statistics from 56 genome-wide significant CAD loci in Nikpay et al. [40], the most recent and largest CAD case-control GWAS meta-analysis to date, to identify 76 candidate genes for CAD (see Methods). Nikpay used 60,801 CAD cases and 123,504 controls from a mix of individuals of mainly European (77%), south (13% India and Pakistan) and east (6% China and Korea) Asian, Hispanic and African American (~4%) descent with genetic variation imputed to a high-density using the 1000 Genomes reference panel. By investigating all SNPs in CAD genes, we aimed to improve detection of smaller polygenic selection signals for the range of functional genic variants and short-range intergenic regulatory variants that would be missed with approaches that only consider genome-wide significant SNPs.

### Signals of positive selection within coronary artery disease loci

We utilised the integrated Haplotype Score (iHS) to estimate positive selection for each SNP underlying CAD genes within each population separately. Because iHS is typically used to detect candidate adaptive SNPs where the selected alleles may not have reached fixation [9], this estimate is well suited for detecting recent signals of selection as opposed to other measures [20]. iHS is also better suited for detecting selection acting on standing variation in polygenic traits [20, 48].

**Fig 1:**
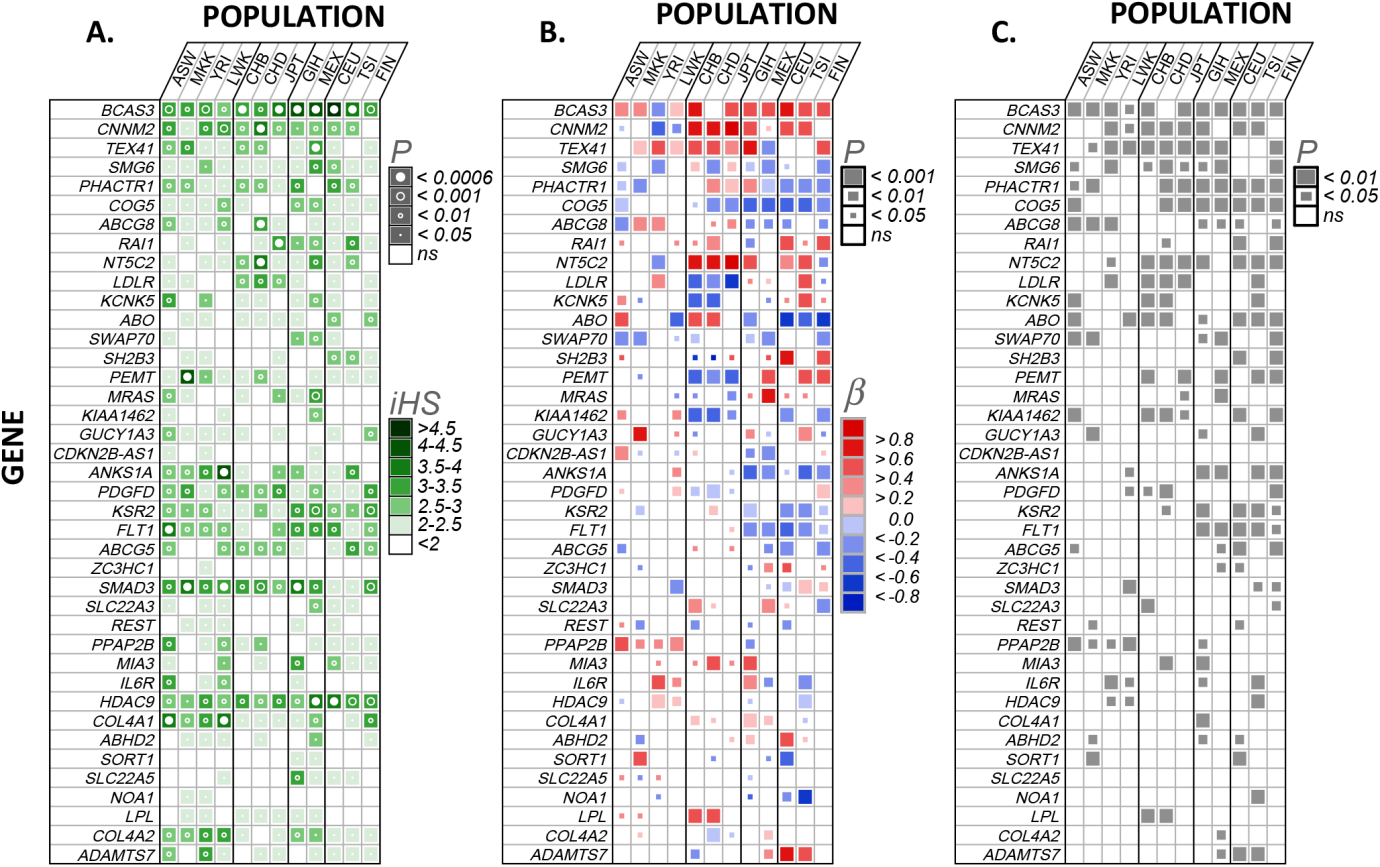
Association of coronary artery disease (CAD) genetic risk and positive signatures of selection in 12 worldwide populations. The 40 of 76 CAD genes investigated are shown that have at least four significant selection-risk associations in Panel B across all 12 populations. **Panel A.** Magnitude and significance of largest positive selection signal (integrated haplotype score, iHS) within each gene-population combination. P values (circles within squares) were obtained from 10000 permutations. Bonferroni corrected p value limit also shown (α = 0.05/76 = 0.000657) with closed circles. **Panel B.** Null hypothesis: no association between CAD genetic risk and positive selection, tested using mixed effects model with SNP estimates of CAD log odds genetic risk and iHS while accounting for gene LD structure as a random effect (first eigenvector from LD matrix per gene). Scaled regression coefficients were obtained directly from regressions, each p value from 10000 permutations. **Panel C.** Null hypothesis: association between genetic risk and positive selection for SNPs within CAD genes no different than non-CAD associated genes. Permuted p values were estimated by comparing each p value in Panel B against 100 nominal p values obtained by randomly choosing (without replacement) 100 non-CAD associated genes of similar size across the genome and using the same mixed effects model setup as described above. **Populations**. Grouped by ancestry, African (ASW, African ancestry in Southwest USA; MKK, Maasai in Kinyawa, Kenya; YRI, Yoruba from Ibadan, Nigeria; LWK, Luhya in Webuye, Kenya), East-Asian (CHB, Han Chinese subjects from Beijing; CHD, Chinese in Metropolitan Denver, Colorado; JPT, Japanese subjects from Tokyo), European (CEU, Utah residents with ancestry from northern and western Europe from the CEPH collection; TSI, Tuscans in Italy; FIN, Finnish in Finland), GIH (Gujarati Indians in Houston, TX, USA), MEX (Mexican ancestry in Los Angeles, CA, USA).

Candidate selection signals were found for many of the 76 CAD genes within each of the 12 worldwide populations (11 HapMap3 populations and Finns; Fig 1A for top 40 based on their association with CAD log odds genetic risk, S1 Fig for all 76). These were defined as ‘peaks’ of significantly elevated iHS scores across SNPs within each gene-population combination, with the apex approximating the likely positional target of positive selection.

The results for the largest iHS score per gene and population (Fig 1A) show that most candidate selection signals were relatively small, but a few larger signals were detected. For example, out of the 912 gene-by-population combinations (S1 Fig), 354 (38%) contained weak-moderate candidate selection signals (significant iHS between 2–3), 84 (9%) contained moderate-strong signals (significant iHS between 3–4), and 6 (0.6%) had very strong signals (significant iHS > 4). The 6 largest candidate signals were found in the following gene-population combinations: *BCAS3* in GIH (iHS = 4.45), MEX (iHS = 4.23) and CEU (iHS = 4.86), *PEMT* in MKK (iHS = 4.24), *ANKS1A* in LWK (iHS = 4.03), and *CXCL12* in JPT (iHS = 4.10), with all iHS p values <0.0001. Six genes (*BCAS3*, *SMG6*, *PDGFD*, *KSR2*, *SMAD3*, *HDAC9*) exhibited candidate selection signals consistently within all populations (Fig 1A), and many genes also contained consistent selection signals for all populations within similar ancestral groups (e.g. African, European etc, Fig 1A).

Within CAD genes, multiple candidate selection signals were sometimes present (particularly within larger genes, within separate linkage disequilibrium (LD)-blocks); these varied between and sometimes within a population. For example, eleven (of the twelve) populations had candidate selection signals in *PHACTR1* introns 4, 7 or 11 (Table 1; see also S2 Fig, comparing cross-population selection signals in *PHACTR1*) that were in separate LD-blocks (see S2 Fig, LD plots). For eight populations, there was a broad and relatively weak set of candidate selection signals in intron 4 (the largest *PHACTR1* intron, ~300kb in length). Intron 4 is also the location of the published CAD index SNP (rs12526453) for *PHACTR1*. Other interesting candidate selection signals present in other CAD genes (S1 Fig) are not discussed here. Such patterns suggest that candidate selection signals are sometimes complex and often do not correspond to the SNPs with largest effect on disease.

**Table 1:** Leading multiple candidate selection signals in *PHACTR1* SNPs.

rs2015764	rs4142300	rs8180558			rs4715043	rs6924689	
12788283 bp	12825772 bp	12919989 bp			12987641 bp	13025819 bp	
MEX, 2.08*	GIH, 3.73***	ASW, 2.43**	LWK, 2.75**	YRI, 2.17*	CHB, 2.26*	CHD, 2.89**	JPT, 3.00**
rs4273688—rs11760186					rs9349549		
13192799–13196011 bp, intron 7					13277029 bp, intron 11		
ASW, 2.38**	LWK, 2.00*	MKK, 2.95**	CHD, 2.05*	GIH, 2.13*	MKK, 2.91**	CEU, 2.71**	TSI, 2.96**

Values include SNP rsID, build 37 base pair chromosomal position, population, absolute integrated Haplotype Score |iHS| and permuted p values (*p<0.05; **p<0.01; ***p<0.001).

Populations: ASW (African ancestry in Southwest USA), MKK (Maasai in Kinyawa, Kenya), YRI (Yoruba from Ibadan, Nigeria), LWK (Luhya in Webuye, Kenya), CHB (Han Chinese subjects from Beijing), CHD (Chinese in Metropolitan Denver, Colorado), JPT (Japanese subjects from Tokyo), CEU (Utah residents with ancestry from northern and western Europe from the CEPH collection), TSI (Tuscans in Italy), GIH (Gujarati Indians in Houston, TX, USA), MEX (Mexican ancestry in Los Angeles, CA, USA).

### Relationship between CAD genetic risk and selection across populations

For each CAD gene within each population, we used a mixed effects linear model to regress SNP-based estimates of CAD log odds genetic risk (ln(OR), obtained from cardiogramplusc4d.org) against iHS selection scores (see Methods). We accounted for LD structure by including the first eigenvector from an LD matrix of correlations (*r*^*2*^) between SNPs within each gene as a random effect.

For a subset of CAD loci, we found significant quantitative associations between disease risk and selection signals and for each of these the direction of this association was often consistent between populations (Fig 1B). Furthermore, when compared to a null distribution of genes selected randomly from the genome, the strength of the CAD log odds versus selection signal at most loci was statistically significant (Fig 1C). Fig 1B shows 40 genes ranked based on those with the most consistent number of significant associations across the 12 populations, with those that showed fewer than four significant associations excluded. Positive and negative associations indicate elevated selection signals present in regions with higher or lower CAD log odds genetic risk, respectively.

In the comparison across populations, directionality of significant selection-risk associations tended to be most consistent for populations within the same ancestral group (Fig 1B). For example, in *PHACTR1*, negative associations were present within all European populations (CEU, TSI, FIN), and in *NT5C2* strong positive associations were present in all East Asian populations (CHB, CHD, JPT). Other negative associations that were consistent across all populations within an ancestry group included five genes in Europeans (*COG5*, *ABO*, *ANKS1A*, *KSR2*, *FLT1*) and four genes (*LDLR*, *PEMT*, *KIAA1462*, *PDGFD*) in East Asians.

Additional consistent positive associations included four genes (*CNNM2*, *TEX41*, *NT5C2*, *MIA3*) in East Asians, three (*BCAS3*, *RAI1*, *KCNK5*) in Europeans, and one (*PPAP2B*) in Africans. In comparison to other ancestral groups, African populations showed fewer significant selection-risk associations (27.9% of all 76-gene x 12-population combinations) than Asians (31.5%) or Europeans (32.8%). Some associations were consistent in all but one population (e.g. *CNNM2*, *ABCG8* in Europeans; *BCAS3*, *KCNK5* in Asians; *CNNM2*, *TEX41* in Africans) or unique to one population within an ancestral group (e.g. *TEX41* in FIN, *COG5* in ASW).

**Fig 2:**
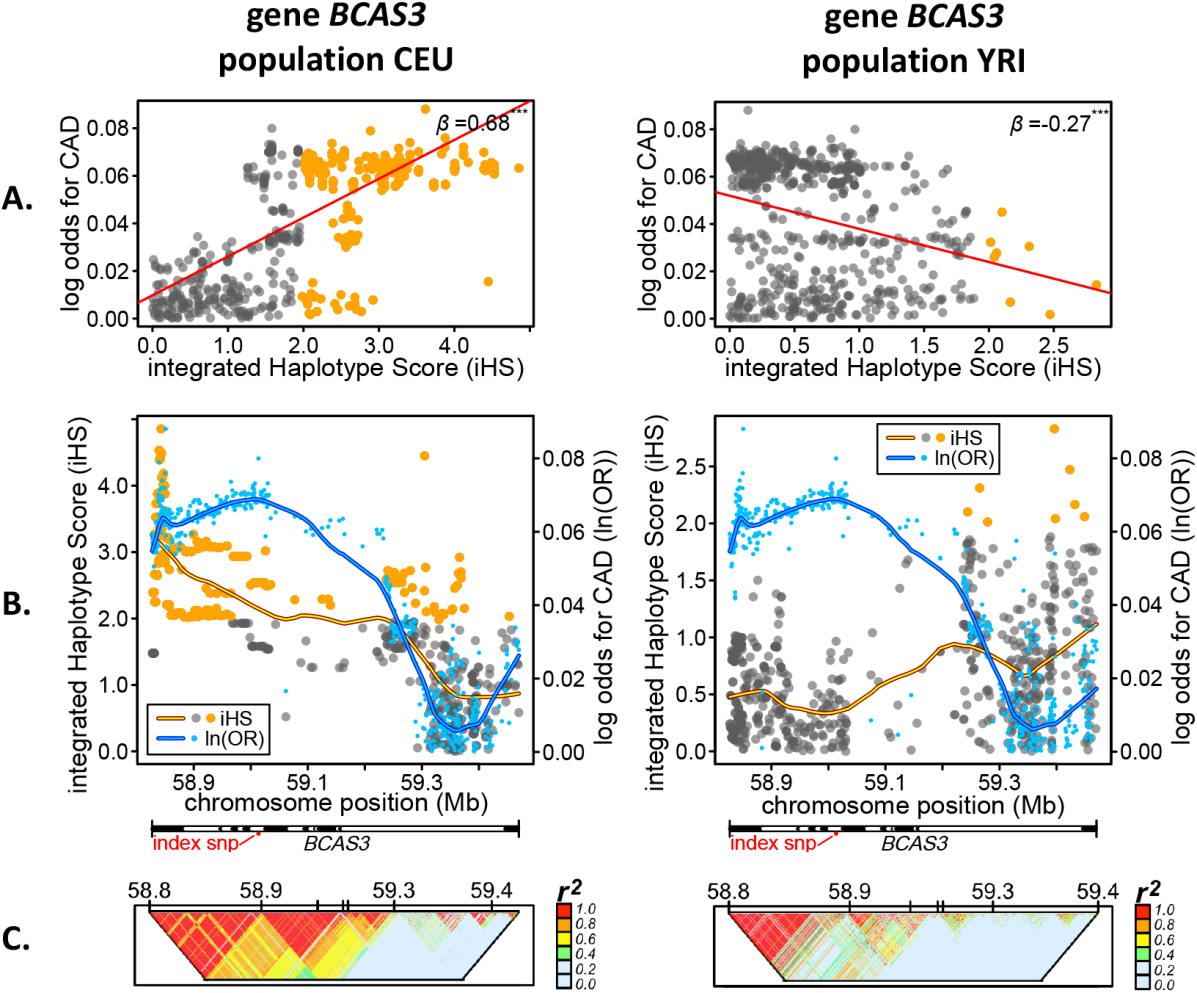
Quantitative links between coronary artery disease risk and selection signals in *BCAS3*. **A.** Correlation between selection signals (iHS) and coronary artery disease (CAD) log odds genetic risk (log odds, ln(OR)), both represented as absolute values. Red line/upper right value, *β* from mixed effects regression. **B.** Base pair positional comparison of selection signals and CAD genetic risk across *BCAS3*. Blue points, CAD log odds values; grey-orange or non-significant-significant points, iHS scores. Horizontal bar shows *BCAS3* gene (and intron) span and location of lead index SNP. Blue/orange lines are smoothed lines estimated with loess function in R. **C.** LD plots, *r*^*2*^. Populations: CEU, Utah residents with ancestry from northern and western Europe from the CEPH collection; YRI, Yoruba from Ibadan, Nigeria.

**Fig 3:**
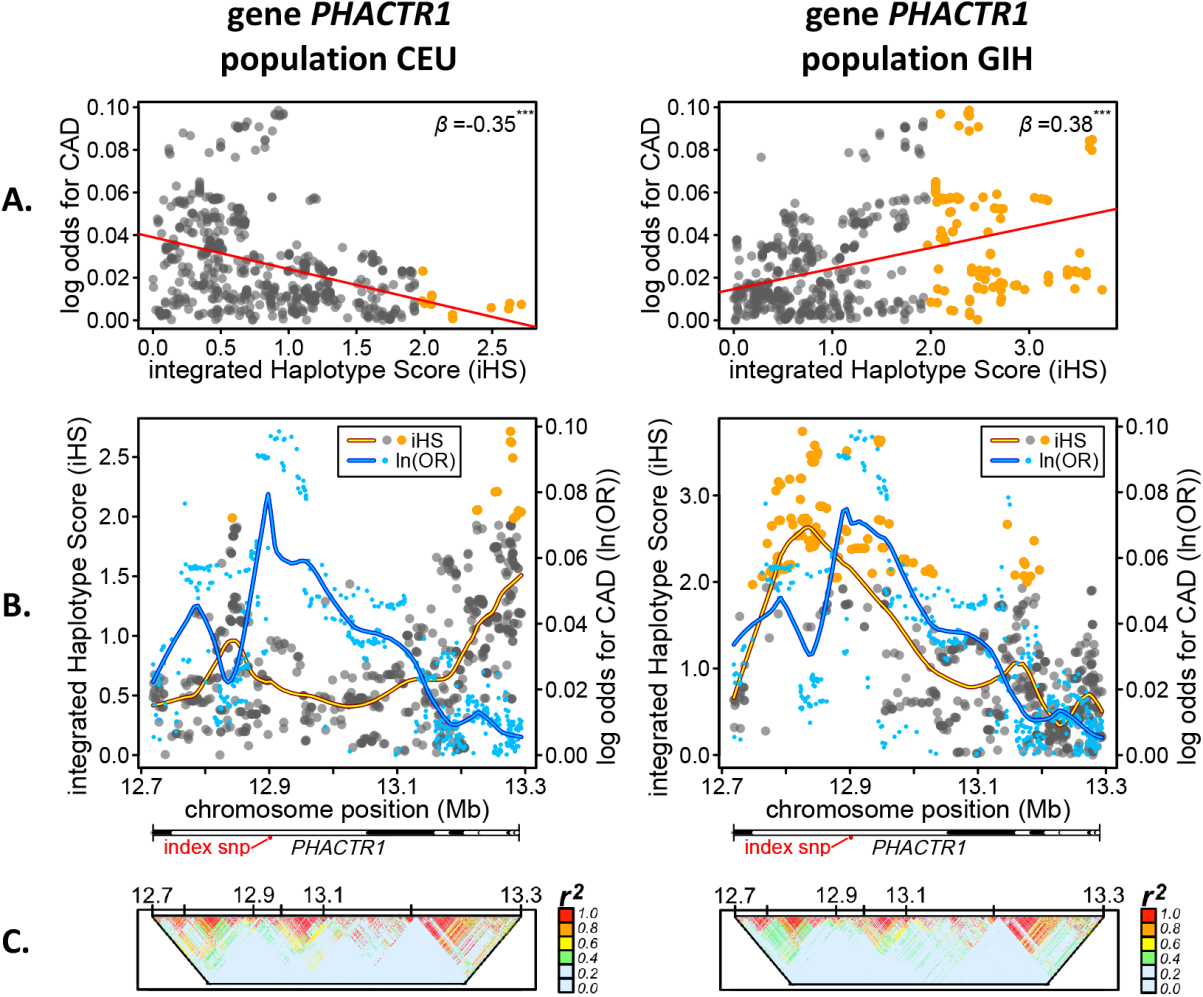
Quantitative links between coronary artery disease risk and selection signals in *PHACTR1*. **A.** Correlation between selection signals (iHS) and coronary artery disease (CAD) log odds genetic risk (ln(OR)), both represented as absolute values. Red line/upper right value, *β* from mixed effects regression. **B.** Base pair positional comparison of selection signals and CAD genetic risk across *PHACTR1*. Blue points, CAD log odds values; grey-orange or non-significant-significant points, iHS scores. Horizontal bar shows *PHACTR1* gene (and intron) spans and location of index SNP if present. **C.** LD plots, *r*^*2*^. Populations: CEU, Utah residents with ancestry from northern and western Europe from the CEPH collection; GIH, Gujarati Indians in Houston, TX, USA.

Below we focus on *BCAS3* (Fig 2) and *PHACTR1* (Fig 3), two of the strongest selection-risk associations which, when adjusting for LD (see Methods), displayed varying directionality between at least two populations.

### Genetic risk of CAD vs positive selection in *BCAS3*

The genetic risks of CAD for variants in *BCAS3* were positively correlated with an extremely large candidate adaptive signal in all European and two of three East Asian populations (Fig 1B). For example in CEU, the largest iHS score was 4.85 and highly significant, and was elevated across most of *BCAS3* (Fig 2B CEU, spanning introns 1–18 and various LD-blocks, Fig 2C), which matched the approximate trends in CAD log odds giving rise to a highly significant positive correlation (Fig 2A CEU). In contrast, in YRI there was no detectable selection signal close to the index SNP (Fig 2B YRI), but weak-moderate signals were present towards the end of *BCAS3* (Fig 2B YRI, introns 18–19, smaller LD-blocks Fig 2C), which also corresponded with lower CAD log odds (Fig 2B, YRI) thus giving rise to a significant negative correlation in Fig 2A.

### Genetic risk of CAD vs positive selection in *PHACTR1*

For all European populations, *PHACTR1* (see CEU example, Fig 3A) selection peaks were typically located within regions of consistently lower CAD log odds (Fig 3B). This contrasted with most other non-European populations where the highest candidate selection peaks were located within regions with elevated CAD log odds (including the index CAD SNP rs12526453, intron 4). The largest selection peak in GIH (Fig 3B) overlapped the CAD log odds peak in *PHACTR1* giving rise to the strong positive association seen in Fig 3A. The two distinctive selection peaks in both CEU and GIH were separated by different LD-blocks (Fig 3C), suggesting that these may have developed independently within *PHACTR1*. Interestingly, the negative association found for the MKK population was due to the location of the selection peaks more closely matching those of the European populations in intron 11 (S2 Fig).

### Enrichment of gene regulatory variants under selection at CAD loci

To establish whether variants with evidence of selection in CAD genes also showed evidence of function, we performed an eQTL scan in 8 HapMap3 populations with matched LCL gene expression. We compared all SNPs in each CAD locus against expression for each focal gene within each population.

**Fig 4:**
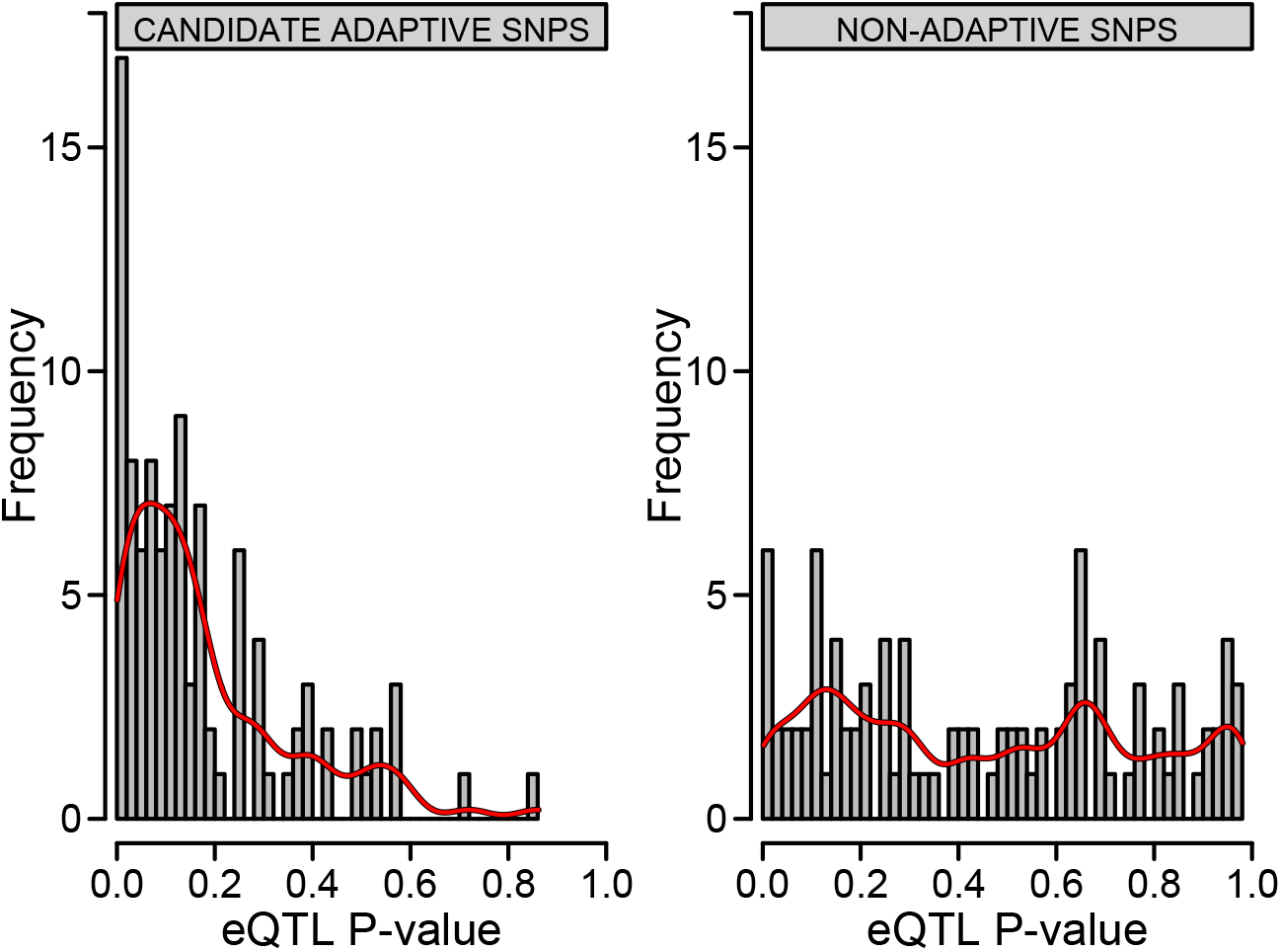
Comparing positive selection with gene regulation. Summary distribution of permuted eQTL p values for SNPs with (left) or without (right) a significant selection signal. SNPs with a significant selection signal (iHS) were chosen by taking the largest significant positive selection signal (if one was present) within each gene-population combination. The same number of SNPs without a significant selection signal were also randomly drawn across all gene-population combinations for comparison. These SNPs were used in an eQTL analysis where they were regressed (including gender as a covariate) against their associated gene probe’s expression.

We found that SNPs with significant integrated Haplotype Scores (iHS) were often also involved in gene regulation, compared to SNPs with non-significant selection scores (Fig 4, Kolmogorov-Smirnov test p value <0.001). To assess which biological pathways were enriched for the highest-ranked genes according to Fig 1B, i.e. those where selection scores were most closely associated with CAD log odds genetic risk, we included the top 10 genes into the Enrichr analysis tool [49] and found that these genes are especially enriched in pathways related to metabolism, focal adhesion and transport of glucose and other sugars. More interestingly, we found connections to reproductive phenotypes in the associations of these genes with pathways, ontologies, cell types and transcription factors. For example, we found links to ovarian steroidogenesis and genes expressed in specific cell types and tissues including the ovary, endometrium and uterus (see S1 Table for Enrichr outputs).

### Enrichment of CAD loci for lifetime reproductive outcomes and antagonistic effects

To test whether CAD genes are directly associated with human lifetime reproductive success (LRS or total number of children born across reproductive lifetimes), a prerequisite for responses to selection, we examined their association with LRS for women in the Framingham Heart Study (FHS). Out of the 76 CAD genes (representing 20,254 SNPs in total; a minimum, average and maximum of 18, 266 and 2121 SNPs tested per gene, respectively), 51 genes contained SNPs that were significantly nominally associated with LRS (p<0.05), 30 genes contained SNPs associated at p<0.01 and 12 genes contained SNPs associated at p<0.001, based on both nominal p values from FaST-LMM and permuted p values (see S2 Table). For example, the most significant associations per gene included rs56152906 in *PPAP2B* (p = 5.23E-06, permuted p<0.0001), rs7896502 in *LIPA* (p = 0.0002, permuted p = 0.0001) and rs2479409 in *PCSK9* (p = 0.0003, permuted p = 0.0001) including a further 9 (*COL4A2*, *FLT1*, *HDAC9*, *KSR2*, *LPA*, *MIA3*, *PDGFD*, *PLG*, *SMAD3*) genes with significant LRS associations at permuted p<0.001. The two previous studies that have investigated genome-wide SNP associations with LRS found associations with similar levels of evidence to our study. For example, the leading SNP in Kosova et al. [50] for completed family size was rs10966811 with p = 5.57E-06. The top two leading SNPs in Aschebrook-Kilfoy et al. [51] for LRS were rs10009124 (p = 7.65E-08) and rs1105228 (p = 2.16E-06).

When we considered these associations using fastBAT that combines SNP associations within a gene (accounting for LD-redundancy) into single gene-level p value, similar results were obtained with 8 genes significantly associated with LRS (e.g. *PPAP2B*, p = 0.0004, permuted p = 0.001, *SMAD3*, p = 0.0061, permuted p = 0.007, *MIA3*, p = 0.008, see S2 Table).

To test the null hypothesis that CAD variation is no more significantly associated with LRS than is variation in the rest of the genome, we used a permutation approach. We sampled 20,254 non-CAD related SNPs (matched within MAF bins to the CAD SNPs) randomly (without replacement) across the genome 100 times. The permuted p value was based on the number of times each random sample of 20,254 non-CAD SNPs shared significantly more associations with LRS than did the 20,254 CAD SNPs. The total sample of randomly selected SNPs (n = 2,025,400) was also compared against the 20,254 CAD SNPs with a Kolmogorov-Smirnov (K-S) test. We found that CAD genetic variation was significantly (p = 9.49E-08 and p = 1.90E-07 based on one- and two-sided K-S tests, respectively; permuted p<0.01) more enriched for LRS compared to the rest of the genome (see S2 Table for other fitness-related traits), providing strong evidence in the FHS for shared fitness effects at CAD loci. This was also the case when we tested this at the gene-level using fastBAT results (permuted p = 0.026, S2 Table).

To test whether effects between CAD loci and LRS were antagonistic, we cross-referenced the genome-wide significant index SNPs for CAD from Nikpay [40] with significant SNPs for LRS from the FaST-LMM analysis. Of the 56 CAD index SNPs in Nikpay [40], 53 were genotyped or imputed in the FHS to a high confidence. In FHS, six of those SNPs (11.3%) were significantly associated with LRS (FaST-LMM p < 0.05), with 5 out of those 6 antagonistic, i.e. the allele that increases LRS also increases risk for CAD (see S3 Table). For example, in *FLT1*, rs9319428-A significantly increases both LRS (ß = 0.041, p = 0.0143) and CAD risk (ß = 0.039, p = 7.13E-05), and similarly, rs2048327-C in *LPA* significantly increases both LRS (ß = 0.041, p = 0.00894) and CAD risk (ß = 0.057, p = 2.46E-09). This suggests that antagonistic effects occur in some loci, but the power to detect and define this for smaller effect variants on LRS is limited in the FHS (e.g. see S3 Fig for power estimates). Compared to the CARDIoGRAMplusC4D study [40] where the 56 genome-wide significant CAD index SNPs were obtained using a meta-sample of ~184,000 individuals, SNP effects on LRS were based on 1,579 women from the FHS. Given that power to detect small effects (i.e. |ß|<~0.3–0.4 or OR <~1.2–1.3) in these studies is poor when *n* is small (i.e. ~1000 individuals [52]) suggests that larger samples of women and men with completed reproduction are needed to test for antagonistic effects comprehensively to avoid false negatives.

We further tested whether SNPs are associated with both LRS and CAD due to potential confounding effects rather than antagonistic pleiotropy, i.e. confounding effects would occur if CAD SNPs influence LRS, which in turn cause significant changes in CAD risk due to physiological, hormonal or social changes related to childbearing/rearing. We tested the association between CAD SNPs and CAD in FHS females, stratified by LRS (see S3 Fig for full analysis). We found no significant effect of LRS modifying SNP effects on CAD (see S3 Fig), which supports the antagonistic pleiotropy hypothesis, however we caution that larger, better powered studies may show some level of attenuation.

Extending this investigation to understand why CAD genes are significantly enriched for LRS, i.e. what possible underlying reproductive processes are contributing, we performed an extensive systematic literature search on the 40 top-ranked genes in Fig 1 and a random set of 20 non-CAD genes. While gene set enrichment had been performed (above) suggesting some connections to reproductive phenotypes, such tools cannot capture the full range of possible effects on multiple fitness traits, some that are themselves rarely tested in other mammalian (non-human) species due to ethical limitations. We found evidence for direct links between CAD genes and fitness (S4 and S5 Tables) including genes associated with reproductive (*PPAP2B*, [53]) or twinning (*SMAD3*, [54]) capacity and number of offspring produced (e.g. *KIAA1462*, [55], *SLC22A5*, [56]). *PHACTR1*, *LPL*, *SMAD3*, *ABO* and *SLC22A5* may contribute to reproductive timing (menarche, menopause) in women [57–59] and animals [60]. Expression of *PHACTR1* [61], *KCNK5* [62], *MRAS* and *ADAMST7* [63] appear to regulate lactation capacity. Some gene deficiencies also cause pregnancy loss (e.g. *LDLR*, [64], *COL4A2*, [65]). Evidence for other pleiotropic links related to fitness included 25 genes that shared links with traits expressed during pregnancy (S4 and S5 Tables), i.e. variation that can negatively influence the health and survival outcomes of both the fetus and mother [66]. For example, a variant of *CDKN2B-AS1* significantly contributes to risk of fetal growth restriction [67], both *FLT1* [68] and *LPL* [69] are significantly differentially expressed in placental tissues from pregnancies with intrauterine growth restriction (IUGR), and preeclampsia and *LDLR*-deficient mice had litters with significant IUGR [70]. A further 29 and 19 genes were linked to traits that can directly influence female and male fertility, respectively (13 influence both) (S4 and S5 Tables). For example, *BCAS3* and *PHACTR1* are highly expressed during human embryogenesis [71, 72], *SWAP70* is intensely expressed at the site of implantation [73], and *PHACTR1* may play a role in receptivity to implantation [74]. For *ABCG8* and *KSR2*, animal models provide further support as gene expression deficiency can cause infertility in females (*ABCG8*, [75]) and males (*KSR2*, [76]).

Pleiotropic connections were also apparent in the classification of specific disorders or from studies investigating single-gene effects. For example, women with polycystic ovarian syndrome (PCOS) have higher rates of infertility due to ovulation failure and modified cardiovascular disease risk factors (i.e. diabetes, obesity, hypertension [77]). While reduced fecundity associated with PCOS might suggest it would not fit the model of antagonistic pleiotropy, some hypothesize that it is an ancient disorder and may have provided a rearing advantage in ancestral food-limited environments [78]. A number of CAD genes in this study (e.g. *PHACTR1*, *LPL*, *PDGFD*, *IL6R*, *CNNM2*) are found differentially expressed in PCOS women [79–83], suggesting possible links between perturbed embryogenesis and angiogenesis. In males, this can be demonstrated with a mutation in *SLC22A5* that causes both cardiomyopathy and male infertility due to altered ability to break down lipids [84, 85]. More generally, many recent studies link altered cholesterol homeostasis with fertility, which is most apparent in patients suffering from hyperlipidemia or metabolic syndrome [86, 87].

For the random set of non-CAD genes that were approximately the same size as the top 20 genes in Fig 1, we were only able to find three (out of 20) with at least one potential link with fitness (S6 Table) using the same systematic literature search further demonstrating the relative abundance of CAD loci effects on fitness earlier in life.

## Discussion

This study identified many candidate adaptive signals suggesting that selection on CAD loci is much more widespread than previously appreciated (also see S1 Discussion). It has previously been suggested [12] and demonstrated [88] that selection on gene expression levels has been an important element of human adaptation in general. We confirm this result for CAD associated loci. Positive selection signals within CAD loci were more likely than random SNPs to be associated with gene expression levels in *cis* (Fig 4).

We found evidence that some of these signals may be a result of selection pressures induced directly by CAD itself. This finding is important for highlighting genes that may have been modified directly by selection on disease phenotypes and also for our general understanding of how quickly human genomes can respond to selection induced by changing environments. Subsequent fitness and biological process analyses and a thorough literature review demonstrated that CAD loci are enriched for lifetime reproductive success in women and also linked to other male and female reproductive phenotypes, which suggests both their potential to respond to natural selection and their possible role via antagonistic pleiotropy in the reproductive tradeoffs that would help to explain why CAD is common in modern humans. While the connection between cardiovascular disease and lifetime parity is not novel (e.g. see [89, 90]), it is not known whether this connection is due to hormonal, physiological, social or selective processes. The current study provides the first evidence for a selective and antagonistic mechanism.

### Coronary artery disease-induced changes to human genomes

One of our most interesting findings was the significant association between selection signals and CAD log odds genetic risk. This approach of integrating genome scans of positive selection with genome-wide genotype-phenotype data has been promoted previously as a tool to uncover biologically meaningful selection signals of recent human adaptation [12, 88] but has rarely been applied. Among the exceptions, Jarvis et al. [91] found a cluster of selection and association signals coinciding on chromosome 3 that included genes *DOCK3* and *CISH*, which are known to affect height in Europeans.

For highly-ranked genes (according to the number of significant associations present within the 12 populations) in Fig 1B such as *BCAS3*, *CNNM2*, *TEX41*, *SMG6* and *PHACTR1*, the consistent overlap between selection and genetic risk of CAD suggests that many of these may have been modified by CAD-linked selective pressures. If so, then two conditions must have been met. Firstly, CAD was present for long enough to be involved in these genetic alterations, an evolutionary process which generally takes thousands of years. Indeed, precursors of CAD (i.e. atherosclerosis) are detectable in very early civilizations [47]. Secondly, the effects of CAD were directly or indirectly expressed during the reproductive period and trait variation was under natural selection due to its effects on reproductive success.

It is only possible for natural selection to directly act on CAD if those outcomes modify individual fitness relative to others in the same population. As outlined in the introduction, this is possible as CAD outcomes (i.e. myocardial infarction) do occur in young adults. However, early-life CAD outcomes are relatively rare, suggesting selection is more likely to operate indirectly on CAD via its risk factors (or other pleiotropically linked traits, discussed below), which provides a more likely explanation for the close associations we found between positive selection and genetic risk. Supporting this, phenotypic selection has been found operating on CAD risk factors [41], suggesting that these selection pressures are still present in modern humans.

Some genes had large signals of selection but showed weak or no consistent overlap with CAD genetic risk. For example *HDAC9* (Histone Deacetylase 9) shows extensive evidence for having undergone recent selection within most populations, especially those of European or Mexican decent, but little or no overlap with CAD risk was evident in most populations. This suggests positive selection has operated on this gene due to its effects on a trait unrelated to CAD, which may not be surprising given *HDAC9*’s broad biological roles (as a transcriptional regulator, cell-cycle progression) and association with other very different phenotypes including ulcerative colitis [92] and psychiatric disorders [93]. This further demonstrates that this approach is useful for separating candidate selection signals important for the disease or phenotype of interest from those that aren’t.

### Pleiotropic effects that establish the genetic foundations of tradeoffs

We found direct evidence in the Framingham Heart Study for shared fitness effects at CAD loci, which were specifically significantly enriched for their effects on female lifetime reproductive success relative to the rest of the genome. This novel finding shows a connection between direct fitness and later disease expressed through CAD loci. An extensive literature review supported this conclusion. All 40 CAD genes from Fig 1 shared at least one (often more) connection with fitness (S4 and S5 Tables). Some appear to directly influence fitness (offspring number, age at menarche, menopause, survival), while many were associated with early-life reproductive traits that are likely to correlate with fitness, including variation in ability to fertilize/conceive or fetal growth, development and survival. This suggests further pleiotropic links between CAD and early-life fitness-related traits. Directly testing for antagonistic effects between fitness and CAD, we found evidence at specific loci for the leading CAD index SNPs, where the allele that significantly increased LRS also significantly increased CAD. We further found no evidence that this link between CAD and LRS was due to confounding (e.g. physiological, hormonal) effects of LRS on CAD risk. While this is promising, the Framingham study is limited in its power to detect small fitness and CAD effects; better powered studies may yet be needed to definitively establish antagonistic effects at all loci. Fitness traits collected on genotyped populations are currently rare, but this is likely to change as more biobank-scale studies come online.

**Fig 5:**
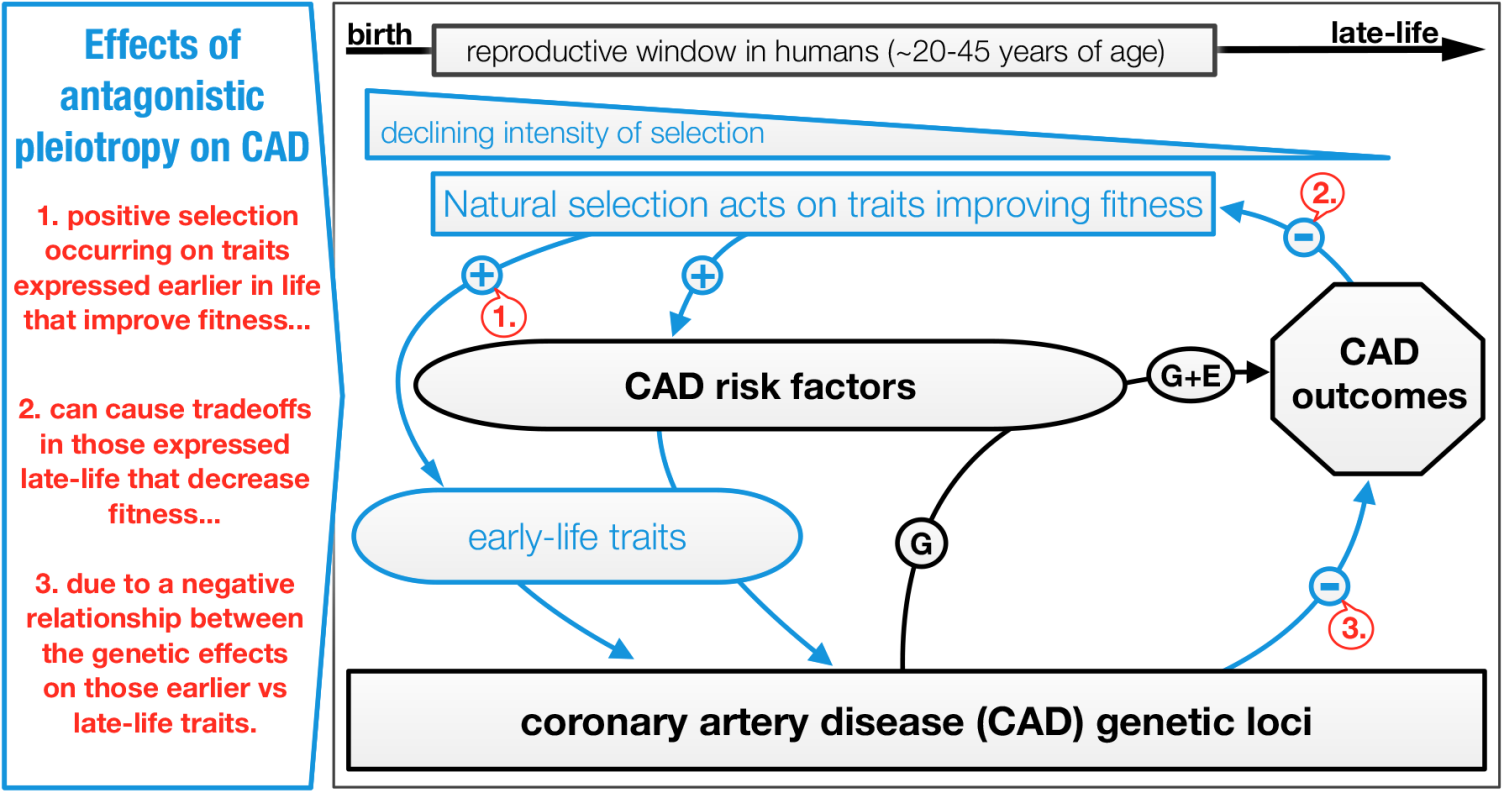
Conceptual figure of potential evolutionary tradeoffs between coronary artery disease (CAD) burden and other phenotypes as a consequence of antagonistic pleiotropy (AP) [42]. As a simple example, AP describes gene effect on two traits (pleiotropy) that oppositely (antagonistic) affect individual fitness at different ages. Selection on that gene conferring a fitness advantage and disadvantage at different ages depends on the size and timing of the effects. An advantage during the ages with the highest probability of reproduction (between~20–45 years of age in humans) would increase fitness (lifetime reproductive success) more than a similarly sized disadvantage at later ages would decrease it. This concept is part of the well-known evolutionary theory of ageing, which describes tradeoffs in energy invested into growth, reproduction and survival [97]. In the figure above, intense natural selection occurring on CAD loci as a result of fitness advantages (+ signs, red text callout box 1.) conferred by genetically correlated risk factors (‘CAD risk factors’ box) or early-life traits (‘early-life traits’ box) trades off with the deleterious effects of these genes on fitness (i.e. CAD burden) later in life (- sign, red text callout box 2.) where the intensity of selection is weak. This occurs because of the negative relationship between genetic effects on early vs late-life traits (- sign, red text callout box 3.), which could help explain the high prevalence and maintenance of CAD in modern human populations. Over shorter timescales, lifetime probability of CAD is modified by a combination of genetic and environmental risk factors (e.g. [98]). There is evidence that such antagonistic effects have operated on CAD loci given: significant associations between CAD genetic risk and selection found (Figs 1 and 2); CAD genes are significantly enriched for lifetime reproductive success (S2 Table) and may also effect other early-life traits known to modify fitness (S4 Table); suggestive evidence was found for an antagonistic relationship between CAD and LRS (S3 Table); phenotypic selection has been found operating on CAD phenotypic risk factors [41].

To facilitate interpretation of selection occurring on early-life traits or CAD phenotypic risk factors that share pleiotropic connections and possible evolutionary tradeoffs with coronary artery disease, we present a conceptual figure (Fig 5). These pleiotropic effects are important because many of them affect traits expressed early in life, some extremely early in life. Any allele that increases reproductive performance enough early in life to more than compensate for a loss of associated fitness late in life will be selected [42]. Such a mechanism has been recently suggested to help explain the maintenance of polymorphic disease alleles in modern human populations [94]. While such tradeoffs have been previously tested for in humans using genotypes, LRS and lifespan (e.g. [95]), there is not yet much evidence that such a mechanism influences human disease. A 2017 study by Rodríguez et al. [96] that used an indirect measure of fitness provides support for antagonistic pleiotropy acting on general early health and later life disease. Our study demonstrates that CAD genes are significantly and directly enriched for fitness with evidence that some of the leading CAD effect SNPs share an antagonistic relationship with fitness through significant positive effects on LRS. This provides support for such a mechanism influencing CAD and may help to explain our vulnerability to this disease.

### Study limitations

There are also some limitations to our approach. We utilized CAD genetic risk estimated from a meta-analysis based on predominantly European (77%) with smaller contributions from south/east Asian (19%), Hispanic and African American (~4%) ancestry [40]. Genetic risk variation for CAD might be different in the un-represented (i.e. Mexican) or less-represented (i.e. African) populations in this meta-analysis. If that were the case, it would reduce the usefulness of comparing selection and risk estimates in those populations. We also saw fewer significant selection-risk associations in the African populations (Fig 1B), however this may be due to selection signals in the African populations being less obvious than those in East Asian and European populations, perhaps due to lesser linkage disequilibrium, as is consistent with results from previous studies [99]. Calculating disease risk and selection variation from populations within the same ancestral group might help resolve this, however it only represents a potential shortcoming for our cross-population analyses and not observations of antagonistic pleiotropy.

### Summary

In this study, we found evidence that natural selection has recently operated on CAD associated variation. By comparing positive selection variation with genetic risk variation at known loci underlying CAD, we were able to identify and prioritize genes that have been the most likely targets of selection related to this disease across diverse human populations. That selection signals and the direction of selection-risk relationships varied among some populations suggests that CAD-driven selection has operated differently in these populations and thus that these populations might respond differently to similar heart disease prevention strategies. The pleiotropic effects that genes associated with CAD have on traits associated with reproduction that are expressed early in life strongly suggests some of the evolutionary reasons for the existence of human vulnerability to CAD.

## Methods

### Defining loci linked to coronary artery disease

We started with the 56 lead index SNPs from Supplementary Table 5 in Nikpay et al. [40] corresponding to 56 CAD loci. When the index SNP was genic, all SNPs within that gene were extracted (using NCBI’s dbSNP) including directly adjacent intergenic SNPs ±5000bp from untranslated regions (UTR) in LD *r*^*2*^>0.7 (with any respective genic SNP). When the index SNP was intergenic, that SNP and other directly adjacent SNPs ±5000bp and in LD>0.7 (with the index SNP) were extracted and combined with SNPs from the respective linked gene listed in Nikpay including SNPs ±5000bp from UTR regions in LD *r*^*2*^>0.7 with that gene. This resulted in SNP lists for 56 genes. To further explore other genes not directly connected with lead index SNPs, but that were within the CAD loci identified by the two most recent CARDIoGRAMplusC4D studies–including Deloukas et al. [39] (i.e. 46 loci and 61 genes listed in their Tables 1–2) and Nikpay et al. [40] (i.e. 10 loci and 15 genes listed in their Table 1)—we extracted SNPs within each of those genes (plus SNPs ±5000bp from UTR regions in LD *r*^*2*^>0.7 with that gene). This resulted in SNP lists for a further 20 genes, bringing the total number of candidate genes for CAD to 76.

The per-SNP log odds (ln(OR)) values for the 76 genes were obtained for the additive model from Nikpay et al. [40] available at http://www.cardiogramplusc4d.org/downloads and used in the analysis described below.

### Preparation of HapMap3 samples

Genotype data (1,457,897 SNPs, 1,478 individuals) were downloaded for 11 HapMap Phase 3 (release 3) populations (http://www.hapmap.org [100]) including: Yoruba from Ibadan, Nigeria (YRI), Maasai in Kinyawa, Kenya (MKK), Luhya in Webuye, Kenya (LWK), African ancestry in Southwest USA (ASW), Utah residents with ancestry from northern and western Europe from the CEPH collection (CEU), Tuscans in Italy (TSI), Japanese from Tokyo (JPT), Han Chinese from Beijing (CHB), Chinese in Metropolitan Denver, Colorado (CHD), Gujarati Indians in Houston, TX, USA (GIH), and Mexican ancestry in Los Angeles, CA, USA (MEX). We also included another HapMap3 population, the Finnish in Finland (FIN) sample (ftp://ftp.fimm.fi/pub/FIN_HAPMAP3 [101]). These data had already been pre-filtered, i.e. SNPs were excluded that were monomorphic, call rate < 95%, MAF<0.01, Hardy-Weinberg equilibrium p<1x10^-6^.

Before phasing and imputation, we performed a divergent ancestry check with flashpca [102] to check accuracy of population assignments, converted SNP data from build 36 to 37 with UCSC LiftOver (https://genome.ucsc.edu/cgi-bin/hgLiftOver), checked strand alignment in Plink v1.9 [103] to ensure all genotypes were reported on the forward strand, and kept only autosomal SNPs. To speed up imputation, data were first pre-phased with Shapeit v2 [104] using the duoHMM option that combines pedigree information to improve phasing and default values for window size (2Mb), per-SNP conditioning sates (100), effective population size (n = 15000) and genetic maps from the 1000 Genomes Phase 3 b37 reference panel (ftp.1000genomes.ebi.ac.uk/vol1/ftp/release/20130502/).

Phased data were imputed in 5 Mb chunks across each chromosome with Impute v2 [105]. We then removed any multiallelic SNPs (insertions, deletions etc) from the imputed data and excluded SNPs with call rate < 95%, HWE p<1x10^-6^ and MAF<1%. The final dataset was then phased with Shapeit v2, and alleles were converted to ancestral and derived states using python script. Ancestral allele states came from 1000 Genomes Project FASTA files and derived 6-primate (human, gorilla, orangutan, chimp, macaque, marmoset) Enredo-Pecan-Ortheus alignment [106] from the Ensembl Compara 59 database [107].

### Estimating signatures of recent selection

*Integrated Haplotype Score (iHS)*: Using the package rehh [108] in R version 3.1.3, per SNP iHS scores were calculated within each population (after excluding non-founders) using methods described previously [9]. iHS could not be calculated for SNPs without an ancestral state, or whose population minor allele frequency is <5%, or for some SNPs that are close to chromosome ends or large regions without SNPs [9]. Rehh was also used to standardize (mean 0, variance 1) iHS values empirically to the distribution of available genome-wide SNPs with similar derived allele frequencies. For analyses in the main text, we considered a SNP to have a candidate selection signal if it had an absolute iHS score > 2, a permuted p value <0.05, and was within a ‘cluster’ of SNPs that also had elevated iHS scores. Although permuting p values is computationally more intensive, it provides more flexibility to detect smaller selection signals that may be incorrectly classified with the more stringent Bonferroni correction that is often applied to these estimates. For the analyses described below, even though we only used iHS estimates for the SNPs defined in the CAD genes (and additional SNPs for permutation purposes), we calculated per-SNP iHS scores genome-wide (rather than locally, i.e. within 1MB regions around focal SNPs), for this provides more accurate estimates because final adjustments are made relative to other genome-wide SNPs of similar sized derived allele frequency classes. P values for iHS scores were permuted based on comparison of nominal p values against 10000 randomly selected estimates from within the same derived allele frequency classes.

### Comparing CAD genetic risk and quantitative selection signals

We first tested the null hypothesis that there is no association between CAD genetic risk and signals of positive selection for CAD genes. For each gene within each population, we used a mixed effects linear model to regress SNP-based estimates of CAD log odds (ln(OR)) genetic risk against selection scores (iHS) resulting in 912 separate regressions. To account for LD structure (and potential confounding of highly correlated SNPs) within each gene, we also included the first eigenvector derived from an LD matrix of correlations (*r*^*2*^) between SNPs within each gene as a random effect. We chose to model LD structure with mixed-effects models rather than LD-prune because for many genes, the SNP samples would have been too small for regression analyses. Also, it would be very difficult to properly capture both selection and the CAD log odds peaks needed to compare these variables. We did however investigate alternative models to validate our approach (i.e. running the same models without the LD structure variable; using smaller multiple LD-pruned subsets of SNPs per gene) with consistent results suggesting our approach was largely robust to LD effects and likelihood of false positive associations. We accounted for multiple testing by permuting p values for each regression based on comparing each nominal p value against 10000 permuted p values derived from shuffling iHS scores.

Genes were then ranked based on the number of significant associations summed across the 12 populations. The 40 genes with at least four or more significant associations are shown in Fig 1B. To illustrate the positional architecture of these selection-risk associations, plots for selected highly-ranked genes are shown in Figs 1 and 2. By demonstrating how CAD genetic risk peaks and valleys correspond to variation in the magnitude of selection scores (iHS), this allowed visual assessment of potential modifications made to the phenotype-genotype map by selective pressures imposed directly or indirectly by CAD. It also helped us localize selection peaks within genes and compare them between populations. Similar peaks suggested similar selection and different peaks suggested local adaptation. This way of presenting the results also allowed us to detect the smaller adaptive shifts in allele frequencies typically expected to underlie selection on polygenic traits.

We then tested a second null hypothesis: that the selection-risk associations using the CAD genes are not unique compared to non-CAD associated loci. For each of the 76 CAD genes, we randomly (without replacement) chose 100 genes of similar length across the genome and performed the same mixed effects regression procedure described above for each gene by population combination using both CAD log odds values from Nikpay et al. [40], iHS scores estimated from the SNP data, and the first LD eigenvector from SNPs within a gene. Permuted p values were derived by comparing the nominal p value for each CAD gene against the 100 null distribution p values from the non-CAD associated genes. Results are shown in Fig 1C.

### Identifying functional targets of selection

To examine whether candidate adaptive signals within each gene corresponded to a gene’s regulatory variation, we regressed SNPs within focal genes and gender against that gene’s probe expression levels, which had previously been quantified in lymphoblastoid cell lines from circulating peripheral blood using Illumina’s Human-6 v2 Expression BeadChip for eight of the 12 populations [109]. Given gene expression in peripheral blood is known to be an important marker for cardiovascular disease, we therefore might expect this cell type a good candidate to search for association between selection signals and regulatory variants important for these genes. The raw gene microarray expression data had previously been normalized on a log2 scale using quantile normalization for replicates of a single individual then median normalization for each population [109]. P values for each SNP-probe association were permuted using 10000 permutations by randomly shuffling gene probes expression. P values were then extracted for the most significant iHS score for each gene-population combination and compared to the same number of p values randomly drawn from different LD blocks underlying SNPs with non-significant iHS scores across each gene-population combination. A Kolmogorov-Smirnov test was used to compare the distribution of p values from each. To examine what biological processes were associated with the top ranked genes from Fig 1, we uploaded the top 10 genes into Enrichr (http://amp.pharm.mssm.edu/Enrichr/) to define associated pathways (i.e. KEGG 2016, kegg.jp/kegg), ontologies (MGI Mammalian phenotypes, informatics.jax.org), cell types (Cancer cell line Encyclopedia, broadinstitute.org/ccle) and transcription factors (ChEA 2015, amp.pharm.mssm.edu/lib/chea.jsp).

### Testing for fitness effects of CAD loci

We tested whether CAD SNPs were directly associated with human fitness. For a trait to evolve, this is one of the main prerequisites, but it also helps demonstrate whether alleles that influence disease also influence reproduction, which in the case of CAD suggests there may be antagonistic trade-offs between early versus late life.

We used the Framingham Heart Study dataset because it has completed reproductive outcomes (lifetime reproductive success (LRS) or number of children ever born), genotypes, pedigree data, cardiovascular outcomes and demographic and socioeconomic data. LRS was derived from clinical questionnaires and further validated with pedigree data. We did not include other datasets for validation here, as it is extremely hard to find others that include all these variables. There were 1,579 women from the Original and Offspring cohorts who had genotypes and all phenotypes available also after excluding non-founders. FHS 500k Affymetrix genotypes were 1000-Genomes imputed using the same pipeline described above bringing the total number of SNPs available (at MAF>1%) to 7,486,901. LRS was adjusted to deal with secular demographic change where data was broken into six groups based on year women were born and divided by the mean reproductive success of women in that group (same as described in [41]).

We examined the association of all SNPs available in the FHS for the 76 CAD genes (20,254 SNPs) with LRS using linear mixed models implemented in FaST-LMM [110] that account for potential confounding effects of genetic similarity by including a *k*-spectral decomposition variable derived from the realized relationship matrix (RRM) of an LD-pruned subset of SNPs. SNPs used for RRM were not in LD with CAD SNPs to avoid proximal contamination. Factors that may affect LRS—education, smoking status, whether the person was born in the US, and estrogen usage (hormone therapy or contraceptive use)—were included as covariates. Permutations with 10,000 iterations were also run for each SNP in order to validate nominal p values obtained directly from FaST-LMM, where permuted p values were based on the number of times nominal p values for 10,000 randomly chosen SNPs (within a similar MAF bin) were greater than the target SNP nominal p value. Bonferroni and FDR adjustment was also applied to p values based on 20,254 tests.

To test the null hypothesis that CAD SNPs are collectively no more enriched for fitness compared to non-CAD SNPs, we randomly sampled without replacement 20,254 non-CAD SNPs (matched within MAF bins to the CAD-SNP sample) 100 times. The permuted p value was based on the number of times (out of 100) that the number of significant p values in the random sample exceeded that for the CAD SNP sample. We also compared the distribution of p values between all randomly chosen SNPs (2,025,400) from this analysis to the 20,254 CAD SNPs with a Kolmogorov-Smirnov test. One- and two-sided tests were run. The one-sided test specifically tested whether the distribution of p values for CAD SNPs was stochastically larger compared to non-CAD SNPs.

We then used fastBAT [111] to test whether CAD is enriched for fitness at the gene-level. fastBAT combines SNP-based summary-level data from GWAS and LD reference data to give locus-based estimates of association. We also ran 100 permutations for each of the 76 genes in order to estimate a permuted p value for each locus. Permuted p values were based on the number of times p values for the 100 randomly chosen similarly-sized genes were greater than that for each CAD gene. We also tested whether CAD genes were collectively more enriched for fitness at the gene-level, relative to non-CAD genes. We randomly chose 76 non-CAD genes of similar size with 300 permutations (sampling without replacement) and asked how many times the number of p values for the non-CAD genes exceeded that of the CAD genes.

Other traits that may influence fitness were also tested (using the same analysis/permutation tests as above) for FHS women including age at first and last birth, interbirth interval, menarche and menopause. Menarche and menopause were derived from questionnaires, while birth timing/spacing were estimated from pedigrees. We did not consider reproductive outcomes for men as that data was only available from pedigrees, which is less reliable than clinical records. Age at first and last birth and interbirth interval were also adjusted for secular demographic changes (same as above).

Alternative simplified models for LRS, AFB and ALB were run in FaST-LMM where fitness measures were not adjusted for temporal effects and no covariates were included. This boosted sample sizes (S2 Table) due to avoiding missing values associated with covariates, however results were largely comparable (S2 Table) suggesting no power gain from unadjusted models.

### Testing for antagonistic effects between fitness and CAD

We only tested for antagonistic effects for LRS as that is the most direct measure of fitness and was the only fitness trait where CAD SNPs were significantly and consistently enriched across (un)adjusted models (S2 Table). To assess whether antagonistic effects were present between LRS and CAD, genome-wide significant CAD index SNPs were taken from Nikpay et al. [40] and cross-referenced with significant LRS SNPs from the FaST-LMM regression results. In an extended analysis (results not shown), we also included any SNP in high-LD (*r*^*2*^> = 0.8) and proximal (±1MB) to the index SNP to boost the number of SNPs available for comparison: results were virtually identical for the significance of LRS and CAD effects and the consistency of antagonistic effects. An antagonistic effect was defined as an allele that significantly increased LRS and significantly increased CAD risk. We also tested whether CAD SNPs were associated with both LRS and CAD due to other confounding (rather than pleiotropic) effects (see S3 Fig for methods and findings).

## Supporting information

S1 Fig Association of coronary artery disease (CAD) risk and genomic signatures of selection in 12 worldwide populations. All 76 genes are shown ranked according to Fig 1B. Boxes show magnitude and significance of largest positive selection signal (integrated haplotype score, iHS) within each gene-population combination. P values (circles within squares) were obtained from 10000 permutations. Bonferroni corrected p value limit also shown (α = 0.05/76 = 0.000657) with closed circles. **Populations**. Grouped by common ancestry, African (ASW, African ancestry in Southwest USA; MKK, Maasai in Kinyawa, Kenya; YRI, Yoruba from Ibadan, Nigeria; LWK, Luhya in Webuye, Kenya), East- Asian (CHB, Han Chinese subjects from Beijing; CHD, Chinese in Metropolitan Denver, Colorado; JPT, Japanese subjects from Tokyo), European (CEU, Utah residents with ancestry from northern and western Europe from the CEPH collection; TSI, Tuscans in Italy; FIN, Finnish in Finland), GIH (Gujarati Indians in Houston, TX, USA), MEX (Mexican ancestry in Los Angeles, CA, USA).
(PDF)

S2 Fig Comparing cross-population candidate selection signals in *PHACTR1*. Per-SNP integrated Haplotype Scores (iHS) plotted by chromosome position within *PHACTR1* (including LD plots below each) for 12 worldwide populations. Permuted p value significance for each score coded by color (grey, non-significant; orange, p < 0.05). Red dashed line indicates position of index SNP for *PHACTR1*. Grey columns in background represent intron spans. Populations are clustered by common ancestry, African (ASW, African ancestry in Southwest USA; MKK, Maasai in Kinyawa, Kenya; YRI, Yoruba from Ibadan, Nigeria; LWK, Luhya in Webuye, Kenya), East-Asian (CHB, Han Chinese subjects from Beijing; CHD, Chinese in Metropolitan Denver, Colorado; JPT, Japanese subjects from Tokyo), European (CEU, Utah residents with ancestry from northern and western Europe from the CEPH collection; TSI, Tuscans in Italy; FIN, Finnish in Finland), GIH (Gujarati Indians in Houston, TX, USA), MEX (Mexican ancestry in Los Angeles, CA, USA).
(PDF)

S3 Fig Testing whether CAD SNPs are associated with both LRS and CAD due to pleiotropy or confounding effects. Confounding effects would occur if coronary artery disease (CAD) SNPs modestly affected lifetime reproductive success (LRS), which in turn caused significant changes in CAD risk due to physiological, hormonal or social changes related to childbearing/rearing [1, 2]. If this was the case, we would expect that the effect of CAD SNPs on CAD should diminish when adjusting for LRS; in the case of pleiotropy, it would not. Grey dots represent regression coefficients (β) for index SNPs on CAD outcomes with (model 2) or without (model 1) stratifying for LRS. β’s are exponeniated coefficients from Cox proportional hazard models that were also adjusted for other potentially confounding effects on CAD (see methods below).
(PDF)

S1 Table Selected Enrichr analysis outputs for top 10-ranked CAD genes with highest genetic risk-selection associations from Fig 1B. Enrichr outputs includes KEGG 2016 Pathways (http://www.kegg.jp/kegg/download/), MGI Mammalian Phenotype Level 3 (http://www.informatics.jax.org/), Cancer Cell Line Encyclopaedia (http://portals.broadinstitute.org/ccle/data/browseData), and ChEA 2015 (http://amp.pharm.mssm.edu/lib/cheadownload.jsp).
(PDF)

S2 Table Testing association of CAD SNPs with human fitness in the Framingham Heart Study women. First three columns give number of individuals available and used in analyses. Four FaST-LMM columns provide summary of leading results including leading and highest ranked SNP(s) (and associated genes). Three fastBAT columns provide leading gene(s). Final four columns provide statistics for testing the Null hypothesis that CAD variation is no more enriched for fitness compared to non-CAD variation found genome-wide.
(PDF)

S3 Table Testing for antagonistic pleiotropy for SNPs with significant effects on lifetime reproductive success (LRS) and coronary artery disease (CAD). *Left table*: provides statistics for CAD index SNPs derived directly from the CARDIoGRAMplusC4D 1000 Genomes-based GWAS meta-analysis (see [1] or http://www.cardiogramplusc4d.org/data-downloads/ for further details of data and variables). *Right table*: provides corresponding FaST-LMM regression statistics of these SNPs on LRS based on Framingham Heart Study women (first six columns), rows correspond to SNPs in the left table. Last four columns test for antagonistic effects between CAD and LRS with the last two providing these tests only when the LRS beta was significant. This shows that when SNPs with significant effects on both LRS and CAD are considered, most (5 of 6, or 83%) were antagonistic, i.e. the allele that increases LRS also increases CAD risk.
(PDF)

S4 Table Pleiotropic links between coronary artery disease (CAD) and early- life fitness-related traits due to shared genetic loci. The table below provides extensive support (143 studies) that antagonistic pleiotropy is likely to be present for CAD genes due to their consistent connections with fitness-related traits expressed early in life. See Fig 5 for discussion and conceptual overview of these potential effects. Fitness-related traits include fertility potential, reproductive outcomes, pregnancy outcomes, fetal growth and survival, i.e. affecting the ability of an organism to reproduce and transfer genes to the next generation. The first 3 columns give CAD gene rank (no.; based on rank of 40 genes from Fig 1B), name and full name. Columns 4–8 provide key details of each study where CAD genes also contribute to traits that influence fitness, including what species that was demonstrated in, what biological process or fitness effects that gene is impacting, what fitness class that effect is likely to impact (e.g. dysfunctional spermatogenesis or embryogenesis will affect male and female fertility, ability to conceive), what the observed genetic effect or mechanism that gene was associated with.
(PDF)

S5 Table Summary of types of pleiotropic connections between coronary artery disease (CAD) and fitness-related traits. Counts are based on S1 Table, ‘fitness class’ column. Most fitness-related traits were related to female potential fertility (29 of 40 genes had these effects) and pregnancy outcomes (25 of 40 genes had these effects). Some genes had broad or specific effects on fitness-related traits. For example, number of fitness classes affected ranged from 6 for *ABO* (had fitness effects across all classes) to 1, for example *CNNM2* (evidence for fitness effects in pregnancy outcomes class).
(PDF)

S6 Table Pleiotropic links between randomly chosen genes and early-life fitness-related traits. Fitness-related traits include fertility potential, reproductive outcomes, pregnancy outcomes, fetal growth and survival, i.e. affecting the ability of an organism to reproduce and transfer genes to the next generation. The first column gives coronary artery disease (CAD) gene (first 20 of 40 CAD genes from Fig 1B/S1 Table). Columns 2–3 give name (abbreviated, full) of randomly chosen genes matched for approximate length for each CAD gene. Columns 4–8 provide key details of each study where random genes also contribute to traits that influence fitness, including what species that was demonstrated in, what biological process or fitness effects that gene is impacting, what fitness class that effect is likely to impact (e.g. dysfunctional spermatogenesis or embryogenesis will affect male and female fertility, ability to conceive), what the observed genetic effect or mechanism that gene was associated with.
(PDF)

S1 Discussion Widespread candidate signals of positive selection on CAD loci. Extended discussion on candidate adaptive signals found on coronary artery disease (CAD) loci in relation to the polygenic model of selection and previous studies examining genomic selection on broader cardiovascular disease loci.
(PDF)
