# Supplementary material for: Genetic loci associated with coronary artery disease harbor evidence of selection and antagonistic pleiotropy
Source: PLoS Genet. 2017 Jun 22;13(6):e1006328. doi: 10.1371/journal.pgen.1006328 (PMC5480811; doi:10.1371/journal.pgen.1006328)
Supplement: S1 Fig — All 76 genes are shown ranked according to Fig 1B. Boxes show magnitude and significance of largest positive selection signal (integrated haplotype score, iHS) within each gene-population combination. P values (circles within squares) were obtained from 10000 permutations. Bonferroni corrected p value limit also shown (α = 0.05/76 = 0.000657) with closed circles. Populations. Grouped by common ancestry, African (ASW, African ancestry in Southwest USA; MKK, Maasai in Kinyawa, Kenya; YRI, Yoruba from Ibadan, Nigeria; LWK, Luhya in Webuye, Kenya), East- Asian (CHB, Han Chinese subjects from Beijing; CHD, Chinese in Metropolitan Denver, Colorado; JPT, Japanese subjects from Tokyo), European (CEU, Utah residents with ancestry from northern and western Europe from the CEPH collection; TSI, Tuscans in Italy; FIN, Finnish in Finland), GIH (Gujarati Indians in Houston, TX, USA), MEX (Mexican ancestry in Los Angeles, CA, USA). (PDF) [file pgen.1006328.s001.pdf]

Supplementary Contents

	Page
**Figure S1.** CAD risk and iHS association for 12 Hapmap populations …………	*S1*
**Figure S2.** Hapmap selection signals for PHACTR1 ………………………………..	*S2-3*
**Table S1.** Enrichr outputs ………………………………………………………………	*S4-5*
**Table S2.** FaST-LMM, fastBAT results …………………………………………………	*S6-7*
**Table S3.** Tests for antagonistic pleiotropy ………………………………………..	*S8*
**Figure S3.** Tests for pleiotropy or confounding …………………………………….	*S9-10*
**Table S4.** Literature search CAD genes (detailed findings) ………………………..	*S11-28*
**Table S5.** Literature search CAD genes (summary) ………………………………..	*S29-30*
**Table S6.** Literature search randomly matched genes ……………………………	*S31-33*
**Supplementary Discussion** …………………………………………………………….	*S34-35*

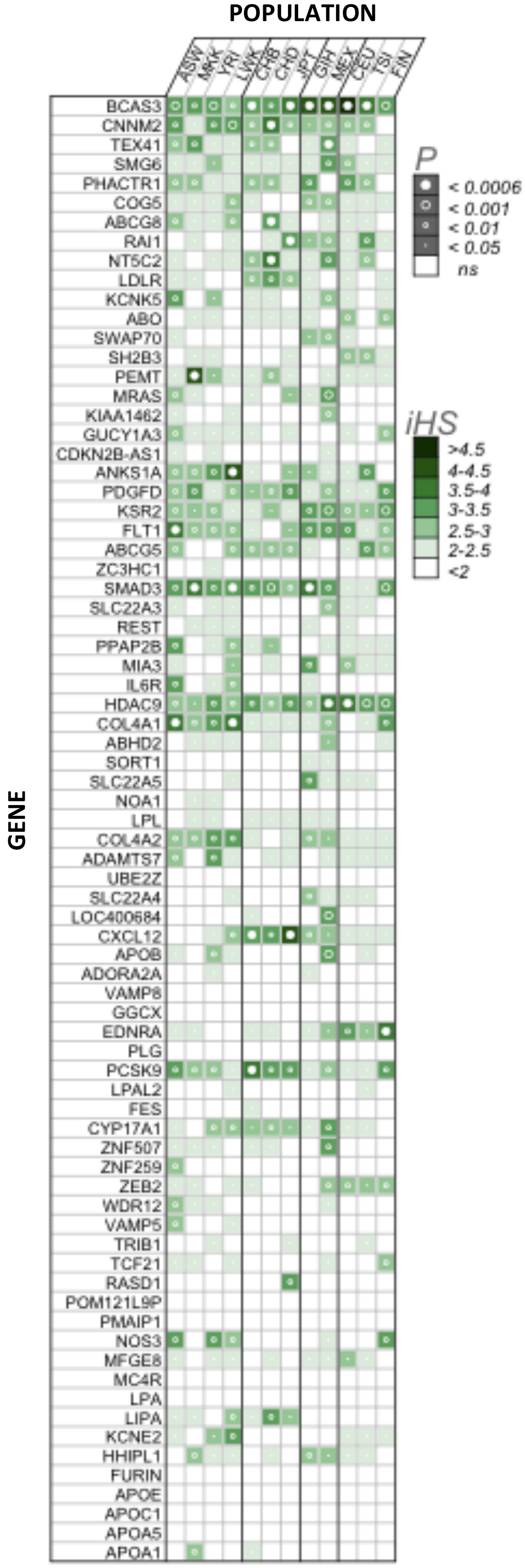

Figure S1. Association of coronary artery disease (CAD) risk and genomic signatures of selection in 12 worldwide populations. All 76 genes are shown ranked according to Fig. 1B. Boxes show magnitude and significance of largest positive selection signal (integrated haplotype score, iHS) within each gene-population combination. P values (circles within squares) were obtained from 10000 permutations. Bonferroni corrected p value limit also shown (α=0.05/76=0.000657) with closed circles. **Populations**. Grouped by common ancestry, African (ASW, African ancestry in Southwest USA; MKK, Maasai in Kinyawa, Kenya; YRI, Yoruba from Ibadan, Nigeria; LWK, Luhya in Webuye, Kenya), East-Asian (CHB, Han Chinese subjects from Beijing; CHD, Chinese in Metropolitan Denver, Colorado; JPT, Japanese subjects from Tokyo), European (CEU, Utah residents with ancestry from northern and western Europe from the CEPH collection; TSI, Tuscans in Italy; FIN, Finnish in Finland), GIH (Gujarati Indians in Houston, TX, USA), MEX (Mexican ancestry in Los Angeles, CA, USA).
