# Supplementary material for: Genetic loci associated with coronary artery disease harbor evidence of selection and antagonistic pleiotropy
Source: PLoS Genet. 2017 Jun 22;13(6):e1006328. doi: 10.1371/journal.pgen.1006328 (PMC5480811; doi:10.1371/journal.pgen.1006328)
Supplement: S2 Fig — Per-SNP integrated Haplotype Scores (iHS) plotted by chromosome position within PHACTR1 (including LD plots below each) for 12 worldwide populations. Permuted p value significance for each score coded by color (grey, non-significant; orange, p < 0.05). Red dashed line indicates position of index SNP for PHACTR1. Grey columns in background represent intron spans. Populations are clustered by common ancestry, African (ASW, African ancestry in Southwest USA; MKK, Maasai in Kinyawa, Kenya; YRI, Yoruba from Ibadan, Nigeria; LWK, Luhya in Webuye, Kenya), East-Asian (CHB, Han Chinese subjects from Beijing; CHD, Chinese in Metropolitan Denver, Colorado; JPT, Japanese subjects from Tokyo), European (CEU, Utah residents with ancestry from northern and western Europe from the CEPH collection; TSI, Tuscans in Italy; FIN, Finnish in Finland), GIH (Gujarati Indians in Houston, TX, USA), MEX (Mexican ancestry in Los Angeles, CA, USA). (PDF) [file pgen.1006328.s002.pdf]

Figure S2

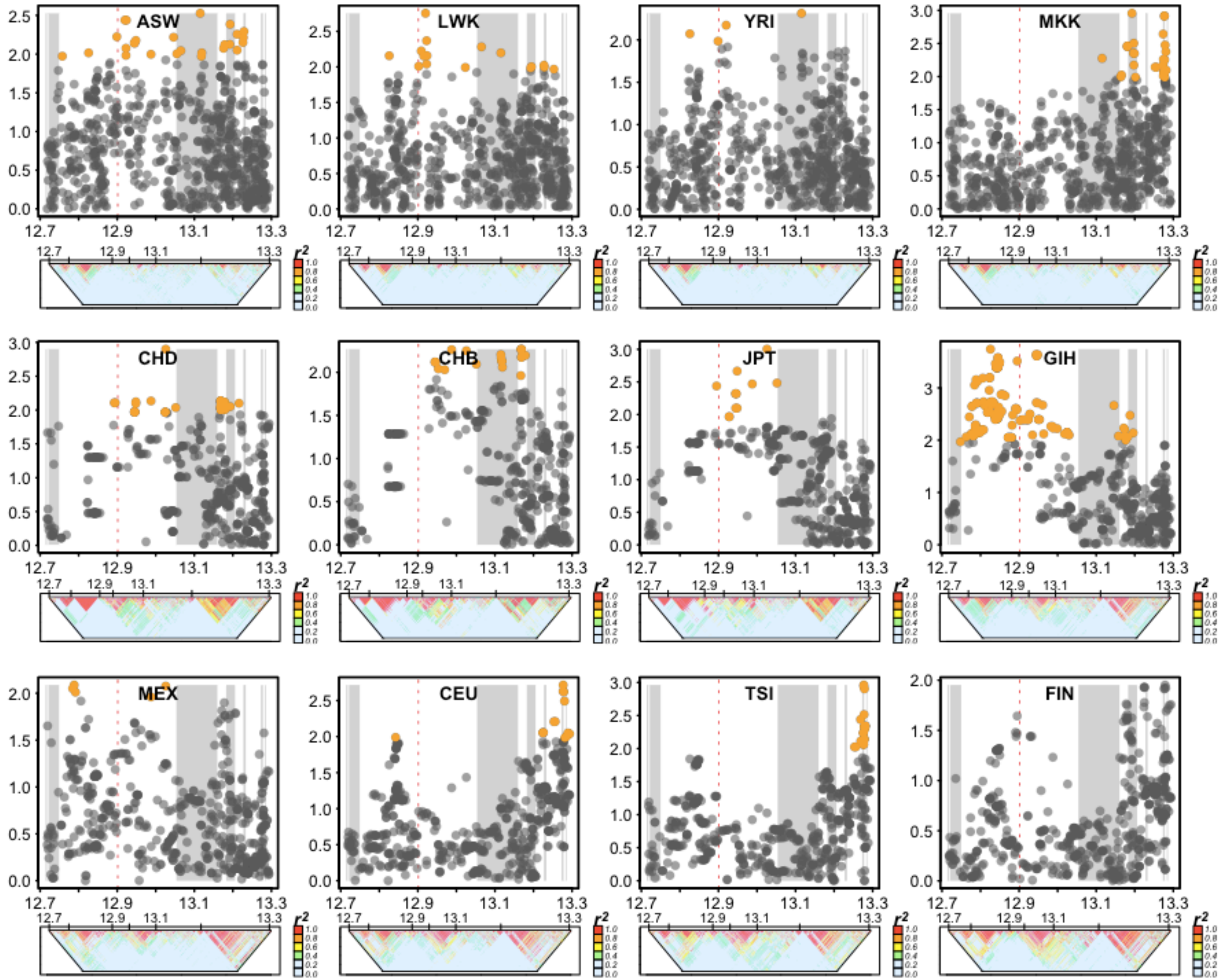
ASW
LWK
YRI
MKK
CHD
CHB
JPT
GIH
MEX
CEU
TSI
FIN
12.7
12.9
13.1
13.3
r2

Figure S2. Comparing cross-population candidate selection signals in *PHACTR1*. Per-SNP integrated Haplotype Scores (iHS) plotted by chromosome position within *PHACTR1* (including LD plots below each) for 12 worldwide populations. Permuted p value significance for each score coded by color (grey, non-significant; orange, $p < 0.05$). Red dashed line indicates position of index SNP for *PHACTR1*. Grey columns in background represent intron spans. Populations are clustered by common ancestry, African (ASW, African ancestry in Southwest USA; MKK, Maasai in Kinyawa, Kenya; YRI, Yoruba from Ibadan, Nigeria; LWK, Luhya in Webuye, Kenya), East-Asian (CHB, Han Chinese subjects from Beijing; CHD, Chinese in Metropolitan Denver, Colorado; JPT, Japanese subjects from Tokyo), European (CEU, Utah residents with ancestry from northern and western Europe from the CEPH collection; TSI, Tuscans in Italy; FIN, Finnish in Finland), GIH (Gujarati Indians in Houston, TX, USA), MEX (Mexican ancestry in Los Angeles, CA, USA).
