# Supplementary material for: Genetic loci associated with coronary artery disease harbor evidence of selection and antagonistic pleiotropy
Source: PLoS Genet. 2017 Jun 22;13(6):e1006328. doi: 10.1371/journal.pgen.1006328 (PMC5480811; doi:10.1371/journal.pgen.1006328)
Supplement: S3 Fig — Confounding effects would occur if coronary artery disease (CAD) SNPs modestly affected lifetime reproductive success (LRS), which in turn caused significant changes in CAD risk due to physiological, hormonal or social changes related to childbearing/rearing [1, 2]. If this was the case, we would expect that the effect of CAD SNPs on CAD should diminish when adjusting for LRS; in the case of pleiotropy, it would not. Grey dots represent regression coefficients (β) for index SNPs on CAD outcomes with (model 2) or without (model 1) stratifying for LRS. β’s are exponeniated coefficients from Cox proportional hazard models that were also adjusted for other potentially confounding effects on CAD (see methods below). (PDF) [file pgen.1006328.s003.pdf]

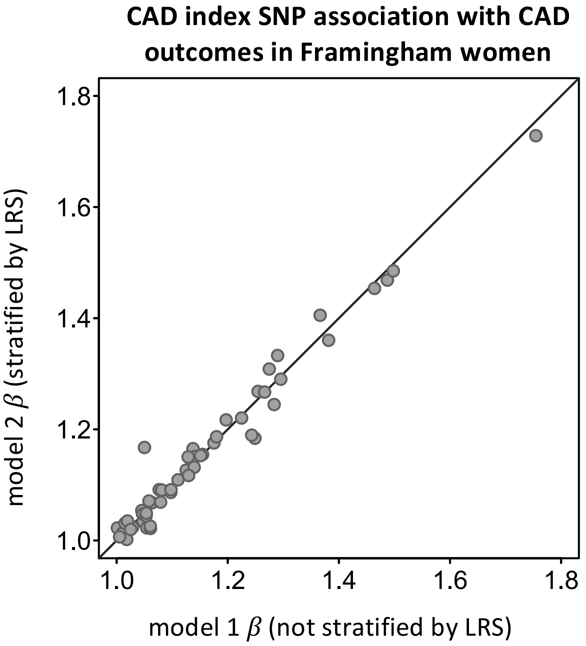

Figure S3. Testing whether CAD SNPs are associated with both LRS and CAD due to pleiotropy or confounding effects. Confounding effects would occur if coronary artery disease (CAD) SNPs modestly affected lifetime reproductive success (LRS), which in turn caused significant changes in CAD risk due to physiological, hormonal or social changes related to childbearing/rearing [1, 2]. If this was the case, we would expect that the effect of CAD SNPs on CAD should diminish when adjusting for LRS; in the case of pleiotropy, it would not. Grey dots represent regression coefficients (β) for index SNPs on CAD outcomes with (model 2) or without (model 1) stratifying for LRS. β's are exponeniated coefficients from Cox proportional hazard models that were also adjusted for other potentially confounding effects on CAD (see methods below).

Methods and results. Here we tested whether CAD index SNPs are significantly associated with lifetime reproductive success (LRS) and coronary artery disease (CAD) due to confounding effects of LRS on CAD or pleiotropic SNP effects on both LRS and CAD. The aim of this was to provide a test of whether the results in Table S3 (i.e. direct tests of antagonistic pleiotropy) were due to pleiotropy or not. Confounding effects would occur if CAD SNPs modestly affected LRS, which in turn caused significant changes in CAD risk due to physiological, hormonal or social changes related to childbearing/rearing [1, 2]. If significant associations between CAD SNPs and either LRS or CAD were due to such confounding, we would expect that the effect of CAD SNPs on CAD should diminish when adjusting for LRS; in the case of pleiotropy, it would not. To test this, we examined the relationship between CAD index SNPs and CAD outcomes for Framingham Heart Study (FHS) women (1227 had complete measures for LRS, CAD, SNPs and covariates) and compared results for models that were not (model 1 β's above) or were (model 2 β's above) stratified by LRS (see Fig. S3 above for summary of results). Beta (β) values are exponeniated coefficients from Cox proportional hazard models that were used to evaluate the association of index SNPs with time to incident CAD events, adjusting for cohort (Original or Offspring), first 5 principal components from LD-thinned genotypes and used age as the time scale. CAD risk factors (prevalent type-2 diabetes, total cholesterol, HDL, systolic BP, smoking status, lipid treatment) were also included as covariates as their effects on CAD outcomes are important and always accounted for in CAD risk analyses. This analysis was performed for 54 CAD index SNPs as listed in Nikpay et al [3] that were also available in the Framingham Heart Study (FHS) imputed genotypes. For CAD outcomes, we used the FHS

definition of CAD, which included recognized/unrecognized myocardial infarction or death from CAD as well as angina pectoris or coronary insufficiency. FHS women with prevalent CAD or <30 years of age at baseline were excluded, and a censoring age of 75 years was also applied. Models (with/without stratifying for LRS) were compared with likelihood ratio tests. There were four ln(*L*) p values that were marginally significant before correction for multiple testing but none after and correlation between model 1 and model 2 SNP effect sizes above were also very similar (r=0.993, Fig. S3). This shows a general lack of significant attenuation of CAD SNP effects on CAD depending on whether analyses were adjusted (stratified) for LRS.

Discussion. Findings from Figure S3 analysis provide support that the results presented in Table S3 (i.e. that CAD index SNPs have antagonistic pleiotropic effects on LRS and CAD) are due to gene pleiotropy rather than confounding effects of childbearing on CAD. As a caveat, these analyses were limited by power. For example, out of the 54 CAD index SNPs examined (these were all highly significantly associated with CAD in the latest CARDIoGRAM meta-analysis, i.e. Nikpay et al [3]), only a few were significantly associated with CAD in the FHS women suggesting that there is low power to detect small effects in the FHS compared to the Nikpay et al study that included ~180000 males and females compared to the 1227 FHS females in our study. We also checked this with power analyses in the R package powerSurvEpi and estimated that on average, in order to test whether a CAD index SNP was significantly associated with CAD with a minimum of 80% power we would need a sample size of ~7600. This was based on the following powerSurvEpi parameters: estimated average CAD index SNP β effect of 1.15 derived from the analyses above, which is slightly higher than the average of 1.07 based on those reported in Tables S3 and S4 in Nikpay et al [3]; postulated power of 80%; average variance in SNPs of ~0.35 estimated from CAD index SNPs in the 1227 FHS women; proportion with CAD outcomes from baseline to 75 years of age that was 10.5% in the 1227 FHS women; alpha set to default of 0.1 for a two-sided test. Therefore, to definitively test for pleiotropic or confounding effects for many SNPs (especially those with smaller effects), larger sample sizes are needed. Currently we do not know of any other larger human datasets with complete measures for LRS, CAD, SNPs and multiple covariates used here. This will become more feasible with larger-scale biobank studies if demographic and reproductive information from electronic health records are also accessible.

References

1. Barrett-Connor, E. and T.L. Bush, *Estrogen and coronary heart disease in women.* JAMA, 1991. **265**(14): p. 1861-7.
2. Beral, V., *Long term effects of childbearing on health.* J Epidemiol Community Health, 1985. **39**(4): p. 343-6.
3. Nikpay, M., et al., *A comprehensive 1000 Genomes-based genome-wide association meta-analysis of coronary artery disease.* Nature Genetics, 2015. **47**(10): p. 1121-+.
