# Supplementary material for: Genetic loci associated with coronary artery disease harbor evidence of selection and antagonistic pleiotropy
Source: PLoS Genet. 2017 Jun 22;13(6):e1006328. doi: 10.1371/journal.pgen.1006328 (PMC5480811; doi:10.1371/journal.pgen.1006328)
Supplement: S1 Table — Enrichr outputs includes KEGG 2016 Pathways (http://www.kegg.jp/kegg/download/), MGI Mammalian Phenotype Level 3 (http://www.informatics.jax.org/), Cancer Cell Line Encyclopaedia (http://portals.broadinstitute.org/ccle/data/browseData), and ChEA 2015 (http://amp.pharm.mssm.edu/lib/cheadownload.jsp). (PDF) [file pgen.1006328.s004.pdf]

Table S1. Selected Enrichr analysis outputs for top 10-ranked CAD genes with highest genetic risk-selection associations from Fig. 1B. Enrichr outputs includes KEGG 2016 Pathways (http://www.kegg.jp/kegg/download/), MGI Mammalian Phenotype Level 3 (http://www.informatics.jax.org/), Cancer Cell Line Encyclopaedia (http://portals.broadinstitute.org/ccle/data/browseData), and ChEA 2015 (http://amp.pharm.mssm.edu/lib/cheadownload.jsp).

PATHWAYS - KEGG 2016 Pathways

Index	Name	P-value	Adjusted p-value	Z-score	Combined score
1	Bile secretion_Homo sapiens_hsa04976	0.0006325	0.008222	-1.77	8.48
2	Ovarian steroidogenesis_Homo sapiens_hsa04913	0.02878	0.07482	-1.85	4.79
3	Fat digestion and absorption_Homo sapiens_hsa04975	0.02374	0.07482	-1.76	4.57
4	Nicotinate and nicotinamide metabolism_Homo sapiens_hsa00760	0.01700	0.07482	-1.75	4.53
5	Aldosterone synthesis and secretion_Homo sapiens_hsa04925	0.04596	0.09556	-1.87	4.39
6	ABC transporters_Homo sapiens_hsa02010	0.02542	0.07482	-1.65	4.28
7	Toxoplasmosis_Homo sapiens_hsa05145	0.06617	0.09558	-1.71	4.01
8	Hepatitis C_Homo sapiens_hsa05160	0.07427	0.09655	-1.61	3.76
9	Pyrimidine metabolism_Homo sapiens_hsa00240	0.05911	0.09558	-1.60	3.75
10	mRNA surveillance pathway_Homo sapiens_hsa03015	0.05145	0.09556	-1.56	3.66

ONTOLOGIES - MGI Mammalian Phenotype Level 3

Index	Name	P-value	Adjusted p-value	Z-score	Combined score
1	IGR1_SKIN	0.002319	0.1055	-1.95	4.38
2	HEYA8_OVARY	0.03735	0.1638	-2.31	4.18
3	OVK18_OVARY	0.003838	0.1055	-1.79	4.03
4	HTK_HAEMATOPOIETIC_AND_LYMPHOID_TISSUE	0.03195	0.1638	-2.13	3.85
5	HS944T_SKIN	0.04174	0.1638	-2.08	3.76
6	MFE296_ENDOMETRIUM	0.04564	0.1638	-2.05	3.71
7	HS746T_STOMACH	0.05146	0.1638	-2.03	3.67
8	WM983B_SKIN	0.05724	0.1638	-1.96	3.54
9	NCIH650_LUNG	0.08805	0.1638	-1.95	3.53
10	TE10_OESOPHAGUS	0.05387	0.1638	-1.94	3.51

CELL TYPES - Cancer Cell Line Encyclopedia

Index	Name	P-value	Adjusted p-value	Z-score	Combined score
1	MP0000003_abnormal_adipose_tissue_	0.01005	0.1061	-2.31	5.19
2	MP0003718_maternal_effect_	0.01434	0.1061	-2.10	4.71
3	MP0005395_other_phenotype_	0.01434	0.1061	-1.88	4.21
4	MP0002139_abnormal_hepatobiliary_system_	0.005946	0.1061	-1.86	4.16
5	MP0009389_abnormal_extracutaneous_pigme_	0.03729	0.1769	-1.87	3.23
6	MP0002168_other_aberrant_phenotype_	0.03869	0.1769	-1.67	2.90
7	MP0001764_abnormal_homeostasis_	0.01658	0.1061	-1.22	2.74
8	MP0005501_abnormal_skin_physiology_	0.05987	0.2395	-1.60	2.29
9	MP0000358_abnormal_cell_content/_	0.07746	0.2680	-1.41	1.86
10	MP0005253_abnormal_eye_physiology_	0.09214	0.2680	-1.37	1.81

TRANSCRIPTION - ChEA 2015

Index	Name	P-value	Adjusted p-value	Z-score	Combined score
1	CTNNB1_20460455_ChIP-Seq_HCT116_Human	4.305e-7	0.00005554	-2.06	20.15
2	TRIM28_17542650_ChIP-ChIP_NTERA2_Human	0.4994	0.5017	-24.98	17.23
3	ESR1_20079471_ChIP-ChIP_T-47D_Human	0.0007167	0.009462	-2.99	13.94
4	FOXP1_22492998_ChIP-Seq_STRATIUM_Mouse	0.005027	0.03393	-4.06	13.72
5	ESR1_22446102_ChIP-Seq_UTERI_Mouse	0.0002684	0.005797	-2.46	12.65
6	FOXA2_19822575_ChIP-Seq_HepG2_Human	0.000003903	0.0002810	-1.20	9.82
7	TFEB_21752829_ChIP-Seq_HELA_Human	0.0003687	0.007099	-1.92	9.50
8	BCL11B_21912641_ChIP-Seq_STHDH STRIUM _Mouse	0.008180	0.04530	-2.86	8.85
9	ARNT_22903824_ChIP-Seq_MCF7_Human	0.0007447	0.009462	-1.83	8.55
10	CIITA_18437201_ChIP-ChIP_Raji B and iDC_Human	0.01114	0.05347	-2.91	8.53
