# Supplementary material for: Genetic loci associated with coronary artery disease harbor evidence of selection and antagonistic pleiotropy
Source: PLoS Genet. 2017 Jun 22;13(6):e1006328. doi: 10.1371/journal.pgen.1006328 (PMC5480811; doi:10.1371/journal.pgen.1006328)

Table S2. Testing association of CAD SNPs with human fitness in the Framingham Heart Study women. First three columns give number of individuals available and used in analyses. Four FaST-LMM columns provide summary of leading results including leading and highest ranked SNP(s) (and associated genes). Three fastBAT columns provide leading gene(s). Final four columns provide statistics for testing the Null hypothesis that CAD variation is no more enriched for fitness compared to non-CAD variation found genome-wide.

	†FHS sample (adjusted) size			*FaST-LMM results				ˆfastBAT results			‡testing Null (CAD not fitness enriched)			
				leading SNP/gene/p		CAD SNPs/genes significant		leading gene			SNP-level	K-S test		gene-level
	adjusted	initial	final	SNP/gene	p	genes	leading SNP	gene	p	CAD genes	permuted p	2-sided	1-sided	p
**lifetime reproductive success**	yes	2226	1579	rs56152906 PPAP2B	5.23E-06	PPAP2B, LIPA, PCSK9, KSR2, PLG, SMAD3, PDGFD, MIA3, COL4A2, LPA, intergenic, HDAC9, FLT1	rs56152906, rs7896502, rs2479409, rs937767, rs4252066, rs1065080, rs117394267, rs17163301, rs9588148, rs3011437, rs1586128, rs146526820, rs9513110	PPAP2B	4.90E-04	PPAP2B, SMAD3, MIA3, PLG, LPA, TCF21, FLT1, NOS3	<0.01	1.90E-07	9.49E-08	0.02
	no	6127	2901	rs9521698 COL4A2	1.30E-04	COL4A2, MIA3, intergenic, HDAC9, NOS3, FLT1, KSR2	rs9521698, rs17163301, rs1586128, rs1726609, rs3800788, rs9508036, rs937767	MIA3	3.24E-04	MIA3, NOS3, SLC22A4, SLC22A5, LIPA, FURIN	<0.01	2.55E-05	1.27E-05	0.04
**age at first birth**	yes	1448	1099	rs117123015 KSR2	3.39E-05	KSR2, SWAP70	rs117123015, rs73408300	ZEB2	1.57E-02	ZEB2, POM121L9P , ADORA2A	0.99	2.29E-01	6.80E-01	0.46
	no	2912	1779	rs786263 TEX41	1.99E-05	TEX41, SMAD3, UBE2Z, HDAC9, PEMT, ADAMTS7, PPAP2B, HHIPL1, ZEB2	rs786263, rs62006053, rs78069344, rs2704287, rs747770, rs2004039, rs11800865, rs141982550, rs7562247	ZEB2	2.77E-03	ZEB2, TEX41, PEMT, CXCL12, RASD1, ADAMTS7	<0.01	4.43E-11	2.21E-11	0.05
**age at last birth**	yes	1448	1099	rs62006053 SMAD3	2.52E-05	SMAD3, PHACTR1, COL4A2, KSR2, HDAC9, NA	rs62006053, rs114969125, rs12018616, rs12305827, rs13245996, rs77032561	ZEB2	6.61E-03	ZEB2, SMAD3, LPL, CXCL12, REST	0.99	3.15E-01	7.36E-01	0.26
	no	2912	1779	rs62006053 SMAD3	4.54E-05	SMAD3, COL4A2, HDAC9, PHACTR1,	rs62006053, rs12018616, rs35710950, rs1008461, rs75374409,	CXCL12	1.96E-03	CXCL12, SMAD3, FES, NT5C2, ADAMTS7,	0.88	7.44E-01	6.14E-01	<0.01

						SLC22A3, ABCG8, NT5C2, FES, LPA, GGCX, KSR2, ADAMTS7	rs56260466, rs145870899, rs78861158, rs41265930, rs115675984, rs524561, rs2004039			FURIN, LPL, ZEB2, RASD1, PEMT				
**inter-birth interval**	no	1954	1290	rs7781749 HDAC9	5.22E-05	PEMT, HDAC9, POM121L9P, COL4A2, NA, BCAS3, WDR12, SMG6	rs3744118, rs12539678, rs3966275, rs76404609, rs35358959, rs111871115, rs6719001, rs1231208	PLG	1.15E-02	PLG, HDAC9, COG5, PCSK9, SLC22A4, SMG6, LPA	0.04	1.29E-01	3.30E-01	0.06
**menarche**	no	1638	1559	rs12428089 COL4A1	2.24E-04	COL4A1	rs12428089	GGCX	2.78E-02	GGCX, VAMP8	0.99	5.79E-01	9.51E-01	0.78
**menopause**	no	4123	2604	rs114162366 PCSK9	3.85E-05	PCSK9, KSR2, HDAC9	rs114162366, rs55730408, rs34966611	APOE	1.74E-02	APOE, APOC1, PHACTR1, PEMT	0.99	3.41E-01	1.71E-01	0.33

† **Framingham Heart Study (FHS) sample size** for females who had reached menopause during the study ensuring completed reproduction. First column indicates whether trait was adjusted for secular demographic change, e.g. in the case of lifetime reproductive success, data was broken into six groups based on year women were born and divided by the mean reproductive success of women in that group. The second to third columns represent reduction in sample sizes due to missing individual data for genotypes and covariates used in the FaST-LMM analysis.

* **FaST-LMM results** are from regressions of CAD SNPs onto each fitness-related trait, using mixed models that accounted for genetic relatedness and population structure and potential confounding covariates (education, smoking, native or foreign-born, estrogen usage). The leading SNP (and associated gene) and Fast-LMM p value are given in the first two columns. While there were many SNPs significant at p < 0.05, for brevity and as an example, SNPs (and their associated genes) with a significant nominal p value from FaST-LMM and permuted p value < 0.001 are shown in the last two columns. Examples are ranked by p value.

ˆ **fastBAT results** were derived with FaST-LMM results and provide locus-level estimates of association. The leading gene and nominal p value is given in the first two columns. The last column provides any genes with significant nominal and permuted p values < 0.05, ranked by p value.

‡ **Testing a Null hypothesis** that CAD-linked SNPs are no more associated with fitness, relative to the rest of the genome. Permutations compared whether there were more significant SNP-fitness associations for 20,254 randomly chosen non-CAD SNPs (matched only for MAF to the CAD-SNP sample) to the 20,254 CAD SNPs. This was repeated (sampling without replacement) 100 times. P values for SNPs came from the FaST-LMM analyses. The first column gives the permuted p value indicating whether the Null was supported or rejected. The second and third columns also tests this by comparing the distribution of p values for all randomly drawn non-CAD SNPs (n=2,025,400) with the 20,254 CAD SNPs using a Kolmogorov Smirnov (K-S) test. Two-sided tests for any difference, one-sided tested whether CAD SNP -log10 p-values were collectively greater than those for non-CAD SNPs. The density plots below compare the distribution of highly significant p-values (p <= 0.001) for the 20,254 CAD SNPs (orange shade) and 2,025,400 randomly drawn (matched within MAF bins) non-CAD SNPs (purple shade). The final column tested this at the gene-level using fastBAT results, i.e. whether the 76 CAD genes were no more significantly enriched than 76 non-CAD genes randomly chosen but matched for approximate gene length. This was repeated 300 times randomly sampling without replacement.

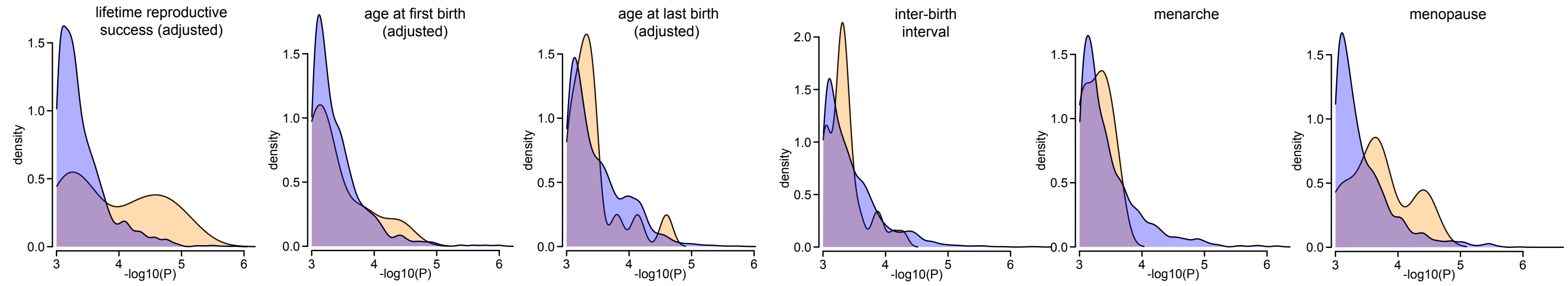

Supplement: S2 Table — First three columns give number of individuals available and used in analyses. Four FaST-LMM columns provide summary of leading results including leading and highest ranked SNP(s) (and associated genes). Three fastBAT columns provide leading gene(s). Final four columns provide statistics for testing the Null hypothesis that CAD variation is no more enriched for fitness compared to non-CAD variation found genome-wide. (PDF) [file pgen.1006328.s005.pdf]
