# Supplementary material for: Genetic loci associated with coronary artery disease harbor evidence of selection and antagonistic pleiotropy
Source: PLoS Genet. 2017 Jun 22;13(6):e1006328. doi: 10.1371/journal.pgen.1006328 (PMC5480811; doi:10.1371/journal.pgen.1006328)
Supplement: S3 Table — Left table: provides statistics for CAD index SNPs derived directly from the CARDIoGRAMplusC4D 1000 Genomes-based GWAS meta-analysis (see [1] or http://www.cardiogramplusc4d.org/data-downloads/ for further details of data and variables). Right table: provides corresponding FaST-LMM regression statistics of these SNPs on LRS based on Framingham Heart Study women (first six columns), rows correspond to SNPs in the left table. Last four columns test for antagonistic effects between CAD and LRS with the last two providing these tests only when the LRS beta was significant. This shows that when SNPs with significant effects on both LRS and CAD are considered, most (5 of 6, or 83%) were antagonistic, i.e. the allele that increases LRS also increases CAD risk. (PDF) [file pgen.1006328.s006.pdf]

Table S3. Testing for antagonistic pleiotropy for SNPs with significant effects on lifetime reproductive success (LRS) and coronary artery disease (CAD). *Left table*: provides statistics for CAD index SNPs derived directly from the CARDIoGRAMplusC4D 1000 Genomes-based GWAS meta-analysis (see [1] or http://www.cardiogramplusc4d.org/data-downloads/ for further details of data and variables). *Right table*: provides corresponding FaST-LMM regression statistics of these SNPs on LRS based on Framingham Heart Study women (first six columns), rows correspond to SNPs in the left table. Last four columns test for antagonistic effects between CAD and LRS with the last two providing these tests only when the LRS beta was significant. This shows that when SNPs with significant effects on both LRS and CAD are considered, most (5 of 6, or 83%) were antagonistic, i.e. the allele that increases LRS also increases CAD risk.

				Nikpay Statistics (additive)							FaST-LMM results (LRS)						Antagonistic effect checks (LRS beta corrected for Nikpay effect allele)			
				b37	effect	non-effect								allele						
index SNP	chr	gene	locus name	position	allele	allele	beta	se_dgc	p_dgc	het_pvalue	beta	se	p value	1	2	MAF	beta (any p value)		beta (p<0.05)	
rs7212798	17	BCAS3	BCAS3	59013488	T	C	-0.0800	0.0142	1.88E-08	2.11E-01	0.0035	0.0165	8.32E-01	C	T	0.1355	-0.0035	antagonistic		
rs12413409	10	CNNM2	CYP17A1-CNNM2-NT5C2	104719096	G	A	0.0752	0.0141	1.07E-07	1.13E-01	-0.0143	0.0159	3.70E-01	A	G	0.0820	0.0143	antagonistic		
rs2252641	2	TEX41	ZEB2-AC074093.1	145801461	C	T	0.0333	0.0096	5.16E-04	7.11E-02	0.0104	0.0159	5.15E-01	C	T	0.4636	0.0104	antagonistic		
rs216172	17	SMG6	SMG6	2126504	G	C	-0.0480	0.0096	5.07E-07	2.00E-01	0.0216	0.0160	1.76E-01	C	G	0.3495	-0.0216	antagonistic		
rs12526453	6	PHACTR1	PHACTR1	12927544	C	G	0.0968	0.0105	2.14E-20	4.58E-01	-0.0073	0.0160	6.49E-01	G	C	0.3245	0.0073	antagonistic		
rs1122608	19	LDLR	LDLR	11163601	G	T	0.0732	0.0110	2.73E-11	7.48E-02	0.0262	0.0159	1.01E-01	T	G	0.2498	-0.0262	-		
rs10947789	6	KCNK5	KCNK5	39174922	T	C	0.0531	0.0111	1.63E-06	5.55E-01	0.0088	0.0160	5.86E-01	C	T	0.2387	-0.0088	-		
rs579459	9	ABO	ABO	136154168	T	C	-0.0730	0.0113	1.14E-10	2.58E-02	0.0117	0.0160	4.66E-01	C	T	0.1889	-0.0117	antagonistic		
rs10840293	11	SWAP70	SWAP70	9751196	A	G	0.0547	0.0096	1.28E-08	1.58E-01	-0.0039	0.0182	8.32E-01	G	A	0.4218	0.0039	antagonistic		
rs3184504	12	SH2B3	SH2B3	111884608	C	T	-0.0642	0.0105	1.03E-09	6.76E-03	-0.0056	0.0198	7.77E-01	T	C	0.4839	0.0056	-		
rs12936587	17	PEMT	RAI1-PEMT-RASD1	17543722	G	A	0.0333	0.0099	8.24E-04	1.98E-01	0.0079	0.0176	6.52E-01	A	G	0.4467	-0.0079	-		
rs9818870	3	MRAS	MRAS	138122122	C	T	-0.0646	0.0137	2.21E-06	4.61E-01	-0.0013	0.0159	9.35E-01	T	C	0.1403	0.0013	-		
rs2505083	10	KIAA1462	KIAA1462	30335122	T	C	-0.0610	0.0095	1.57E-10	2.36E-01	0.0035	0.0181	8.46E-01	C	T	0.3981	-0.0035	antagonistic		
rs7692387	4	GUCY1A3	GUCY1A3	156635309	G	A	0.0679	0.0117	7.35E-09	7.64E-01	0.0155	0.0160	3.32E-01	A	G	0.1805	-0.0155	-		
rs3217992	9	CDKN2B-AS1	CDKN2BAS1	22003223	C	T	-0.1283	0.0094	1.03E-42	3.25E-04	0.0173	0.0164	2.94E-01	T	C	0.3939	-0.0173	antagonistic		
rs17609940	6	ANKS1A	ANKS1A	35034800	G	C	0.0276	0.0125	2.65E-02	3.59E-01	0.0237	0.0160	1.40E-01	C	G	0.1857	-0.0237	-		
rs974819	11	PDGFD	PDGFD	103660567	C	T	-0.0634	0.0100	2.44E-10	2.17E-01	0.0070	0.0163	6.66E-01	T	C	0.3082	-0.0070	antagonistic		
rs11830157	12	KSR2	KSR2	118265441	T	G	-0.0347	0.0098	3.88E-04	1.39E-01	0.0220	0.0184	2.32E-01	G	T	0.3703	-0.0220	antagonistic		
rs9319428	13	FLT1	FLT1	28973621	G	A	-0.0396	0.0100	7.13E-05	3.90E-01	0.0415	0.0169	1.43E-02	A	G	0.2877	-0.0415	antagonistic	-0.0415	antagonistic
rs6544713	2	ABCG5	ABCG5-ABCG8	44073881	C	T	-0.0515	0.0105	8.88E-07	5.40E-02	0.0161	0.0163	3.23E-01	T	C	0.3303	-0.0161	antagonistic		
rs11556924	7	ZC3HC1	ZC3HC1	129663496	C	T	0.0726	0.0111	5.34E-11	8.44E-02	0.0057	0.0160	7.21E-01	T	C	0.3797	-0.0057	-		
rs56062135	15	SMAD3	SMAD3	67455630	C	T	0.0697	0.0119	4.52E-09	6.68E-01	-0.0109	0.0171	5.24E-01	T	C	0.2080	0.0109	antagonistic		
rs17087335	4	REST	REST-NOA1	57838583	G	T	-0.0608	0.0111	4.59E-08	1.14E-01	-0.0140	0.0159	3.79E-01	T	G	0.1811	0.0140	-		
rs17114036	1	PPAP2B	PPAP2B	56962821	A	G	0.1231	0.0168	2.22E-13	6.45E-02	0.0199	0.0161	2.17E-01	G	A	0.0940	-0.0199	-		
rs17464857	1	MIA3	MIA3	222762709	T	G	0.0573	0.0140	4.18E-05	4.71E-01	0.0142	0.0159	3.69E-01	G	T	0.1457	-0.0142	-		
rs4845625	1	IL6R	IL6R	154422067	C	T	-0.0507	0.0092	3.93E-08	6.40E-01	0.0018	0.0161	9.09E-01	T	C	0.4178	-0.0018	antagonistic		
rs2023938	7	HDAC9	HDAC9	19036775	T	C	-0.0591	0.0155	1.36E-04	2.97E-01	-0.0436	0.0159	6.24E-03	C	T	0.0892	0.0436	-	0.0436	-
rs9515203	13	COL4A1	COL4A1-COL4A2	111049623	T	C	0.0711	0.0116	9.33E-10	1.39E-01	0.0176	0.0184	3.39E-01	C	T	0.1985	-0.0176	-		
rs8042271	15	ABHD2	MFGE8-ABHD2	89574218	A	G	-0.0967	0.0176	3.68E-08	1.86E-01	-0.0264	0.0162	1.03E-01	A	G	0.0470	-0.0264	antagonistic		
rs602633	1	SORT1	SORT1	109821511	G	T	0.0960	0.0115	6.97E-17	7.33E-03	-0.0073	0.0163	6.53E-01	T	G	0.2171	0.0073	antagonistic		
rs264	8	LPL	LPL	19813180	G	A	0.0583	0.0132	1.06E-05	9.78E-01	0.0188	0.0160	2.40E-01	A	G	0.1355	-0.0188	-		
rs7173743	15	ADAMTS7	ADAMTS7	79141784	T	C	0.0755	0.0093	5.55E-16	2.12E-01	0.0188	0.0163	2.48E-01	C	T	0.4627	-0.0188	-		
rs46522	17	UBE2Z	UBE2Z	46988597	C	T	-0.0398	0.0093	1.84E-05	6.44E-01	0.0028	0.0159	8.60E-01	C	T	0.4658	0.0028	-		
rs273909	5	SLC22A4	SLC22A4-SLC22A5	131667353	A	G	-0.0559	0.0146	1.24E-04	4.79E-01	0.0027	0.0166	8.69E-01	G	A	0.0726	-0.0027	antagonistic		
rs12976411	19	LOC400684	ZNF507-LOC400684	32882020	A	T	0.0478	0.0183	9.12E-03	1.03E-01	-0.0010	0.0164	9.50E-01	T	A	0.0385	0.0010	antagonistic		
rs2047009	10	CXCL12	CXCL12	44539913	T	G	-0.0613	0.0092	2.75E-11	1.54E-01	0.0041	0.0159	7.97E-01	T	G	0.4769	0.0041	-		
rs515135	2	APOB	APOB	21286057	C	T	0.0675	0.0122	3.09E-08	9.42E-01	0.0159	0.0160	3.23E-01	T	C	0.1979	-0.0159	-		
rs1878406	4	EDNRA	EDNRA	148393664	C	T	-0.0604	0.0125	1.24E-06	3.59E-02	-0.0137	0.0160	3.93E-01	T	C	0.1399	0.0137	-		
rs4252120	6	PLG	PLG	161143608	T	C	0.0329	0.0112	3.32E-03	8.71E-01	-0.0468	0.0159	3.32E-03	C	T	0.2967	0.0468	antagonistic	0.0468	antagonistic
rs11206510	1	PCSK9	PCSK9	55496039	T	C	0.0745	0.0133	2.34E-08	5.58E-01	-0.0012	0.0159	9.39E-01	C	T	0.1932	0.0012	antagonistic		
rs17514846	15	FES	FURIN-FES	91416550	C	A	-0.0509	0.0100	3.10E-07	2.42E-01	-0.0372	0.0344	2.79E-01	A	C	0.4834	0.0372	-		
rs6725887	2	WDR12	WDR12	203745885	T	C	-0.1329	0.0155	9.51E-18	3.93E-01	-0.0214	0.0159	1.78E-01	C	T	0.1308	0.0214	-		
rs1561198	2	VAMP5	VAMP5-VAMP8-GGCX	85809989	C	T	-0.0584	0.0095	6.37E-10	4.03E-01	0.0216	0.0161	1.79E-01	T	C	0.4663	-0.0216	antagonistic		
rs2954029	8	TRIB1	TRIB1	126490972	A	T	0.0439	0.0093	2.61E-06	2.42E-01	0.0169	0.0162	2.96E-01	T	A	0.4396	-0.0169	-		
rs12190287	6	TCF21	TCF21	134214525	C	G	0.0580	0.0177	1.07E-03	1.24E-09	-0.0435	0.0176	1.33E-02	G	C	0.3670	0.0435	antagonistic	0.0435	antagonistic
rs180803	22	POM121L9P	POM121L9P-ADORA2A	24658858	T	G	-0.1809	0.0283	1.64E-10	6.68E-01	-	-	-	-	-	0.0092	-	-	-	-
rs663129	18	PMAIP1	PMAIP1-MC4R	57838401	G	A	-0.0582	0.0105	3.20E-08	5.95E-01	0.0106	0.0160	5.06E-01	A	G	0.2172	-0.0106	antagonistic		
rs3918226	7	NOS3	NOS3	150690176	C	T	-0.1333	0.0221	1.69E-09	1.94E-01	0.0364	0.0175	3.84E-02	T	C	0.0278	-0.0364	antagonistic	-0.0364	antagonistic
rs2048327	6	LPA	SLC22A3-LPAL2-LPA	160863532	T	C	-0.0575	0.0096	2.46E-09	8.72E-03	0.0418	0.0160	8.94E-03	C	T	0.3648	-0.0418	antagonistic	-0.0418	antagonistic
rs11203042	10	LIPA	LIPA	90989109	C	T	-0.0355	0.0092	1.22E-04	3.10E-01	-0.0238	0.0165	1.50E-01	T	C	0.4519	0.0238	-		
rs9982601	21	KCNE2	KCNE2	35599128	C	T	-0.1098	0.0148	1.33E-13	5.43E-02	-0.0084	0.0161	6.02E-01	T	C	0.1369	0.0084	-		
rs2895811	14	HHIPL1	HHIPL1	100133942	T	C	-0.0408	0.0095	1.86E-05	3.75E-02	-0.0209	0.0202	3.01E-01	C	T	0.3636	0.0209	-		
rs445925	19	APOC1	ApoE-ApoC1	45415640	G	A	0.0858	0.0187	4.23E-06	5.30E-02	0.0169	0.0204	4.09E-01	A	G	0.0356	-0.0169	-		
rs9326246	11	APOA1	ZNF259-APOA5-APOA1	116611733	G	C	-0.0458	0.0151	2.46E-03	2.56E-02	-0.0253	0.0159	1.12E-01	C	G	0.0739	0.0253	-		
																			**5 of 6 (83%) antagonistic**	

References

1. Nikpay, M., et al., *A comprehensive 1000 Genomes-based genome-wide association meta-analysis of coronary artery disease.* Nature Genetics, 2015. **47**(10): p. 1121-+.
