# Supplementary material for: Genetic loci associated with coronary artery disease harbor evidence of selection and antagonistic pleiotropy
Source: PLoS Genet. 2017 Jun 22;13(6):e1006328. doi: 10.1371/journal.pgen.1006328 (PMC5480811; doi:10.1371/journal.pgen.1006328)
Supplement: S4 Table — The table below provides extensive support (143 studies) that antagonistic pleiotropy is likely to be present for CAD genes due to their consistent connections with fitness-related traits expressed early in life. See Fig 5 for discussion and conceptual overview of these potential effects. Fitness-related traits include fertility potential, reproductive outcomes, pregnancy outcomes, fetal growth and survival, i.e. affecting the ability of an organism to reproduce and transfer genes to the next generation. The first 3 columns give CAD gene rank (no.; based on rank of 40 genes from Fig 1B), name and full name. Columns 4–8 provide key details of each study where CAD genes also contribute to traits that influence fitness, including what species that was demonstrated in, what biological process or fitness effects that gene is impacting, what fitness class that effect is likely to impact (e.g. dysfunctional spermatogenesis or embryogenesis will affect male and female fertility, ability to conceive), what the observed genetic effect or mechanism that gene was associated with. (PDF) [file pgen.1006328.s007.pdf]

Table S4. Pleiotropic links between coronary artery disease (CAD) and early-life fitness-related traits due to shared genetic loci. The table below provides extensive support (143 studies) that antagonistic pleiotropy is likely to be present for CAD genes due to their consistent connections with fitness-related traits expressed early in life. See Fig. 5 for discussion and conceptual overview of these potential effects. Fitness-related traits include fertility potential, reproductive outcomes, pregnancy outcomes, fetal growth and survival, i.e. affecting the ability of an organism to reproduce and transfer genes to the next generation. The first 3 columns give CAD gene rank (no.; based on rank of 40 genes from Fig. 1B), name and full name. Columns 4-8 provide key details of each study where CAD genes also contribute to traits that influence fitness, including what species that was demonstrated in, what biological process or fitness effects that gene is impacting, what fitness class that effect is likely to impact (e.g. dysfunctional spermatogenesis or embryogenesis will affect male and female fertility, ability to conceive), what the observed genetic effect or mechanism that gene was associated with.

no.	CAD gene	full name	species	fitness effects	fitness class*	observed genetic effect or mechanism	ref
**1**	***BCAS3***	Breast Carcinoma Amplified Sequence 3	human/ mouse	embryogenesis	female potential fertility	*BCAS3* highly expressed in developing oocytes	[1]
	BCAS3		mouse	embryogenesis	female potential fertility	*BCAS3* significantly up-regulated in developmentally incompetent mouse oocytes	[2]
**2**	***CNNM2***	Cyclin And CBS Domain Divalent Metal Cation Transport Mediator 2	human	pregnancy-related blood pressure	pregnancy outcomes	*CNNM2* significantly differentially expressed	[3]
	CNNM2		mouse	pregnancy complications, hypoxia	pregnancy outcomes	*CNNM2* significantly down-regulated (-2.5 fold change) during pregnancy	[4]
**3**	***TEX41***	Testis Expressed 41 (Non- Protein Coding)	human	fetal IUGR, developmental delays	pregnancy outcomes	triplication involving *TEX41* causes IUGR	[5]
**4**	***SMG6***	Nonsense Mediated MRNA Decay Factor	mouse	altered embryogenesis	female potential fertility	*SMG6* essential for normal embryogenesis based on gene knock-down study	[6]
**5**	***PHACTR1***	Phosphatase And Actin Regulator 1	human	reproductive timing	reproductive outcomes	*PHACTR1* genetic variation	[7]
	PHACTR1		human	oocyte function	female potential fertility	*PHACTR1* highly significantly expressed	[8]
	PHACTR1		human	placental inflammatory responses	pregnancy outcomes	*PHACTR1* significantly down-regulated	[9]
	PHACTR1		human	endometrium implantation receptivity	female potential fertility	*PHACTR1* 8-fold significantly up-regulated	[10]
	PHACTR1		mouse	uterus functioning	female potential fertility	*PHACTR1* significant 1.4-1.9 fold change	[11]
	PHACTR1		rat	lactation	reproductive outcomes	*PHACTR1* significantly expressed (4.7 fold change) in mammary tissues	[12]

**6**	***COG5***	Component of Oligomeric Golgi Complex 5	*Drosophila*	spermatogenesis	male potential fertility	*COG5* expression required for normal spermatogenesis	[13]
	COG5		human	intrauterine growth	pregnancy outcomes	*COG5* expression required for normal fetal growth	[14]
**7**	***ABCG8***	ATP-Binding Cassette, Sub-Family G, Member 8	mouse	infertility	female potential fertility	Knockout mice deficient *Abcg8* are infertile	[15]
	ABCG8		human	fetal distress, asphyxial events, intrauterine death	pregnancy outcomes	*ABCG8* involved in intrahepatic cholestasis of pregnancy (ICP), enterohepatic circulation, specifically for exportation of cholesterol	[16]
**8**	***RAI1***	Retinoic Acid Induced 1	human/ mouse	growth retardation, embryonic-postnatal development	pregnancy outcomes	knock-out mouse model for Smith-Magenis syndrome shows involvement of *RAI1*	[17]
	RAI1		mouse	growth retardation, impaired motor and sensory coordination, smaller litter size (direct reproductive fitness)	pregnancy outcomes	Transgenic mice over-expressing *RAI1* have developmentally impaired offspring	[18]
**9**	***NT5C2***	Nucleotidase, Cytosolic II	human	female reproduction	female potential fertility	*NT5C2* is over-expressed in fallopian tube, uterine endometrium, endocervix, ectocervix	[19]
	NT5C2		human	fetal growth, birthweight, postnatal growth & metabolism	pregnancy outcomes	*NT5C2* genetic variation affects birthweight	[20]
**10**	***LDLR***	Low Density Lipoprotein Receptor	human/ mouse	IUGR in offspring of *LDLR*-/- mice. Childhood obesity.	pregnancy outcomes	*LDLR* involved in fetal/offspring growth	[21]
	LDLR		human	Placental regulation of cholesterol	pregnancy outcomes	Maternal lipid profile affecting placental protein expression of *LDLR*	[22]
	LDLR		mouse	Placental regulation of cholesterol	pregnancy outcomes	LDLR involved in maternal-fetal transfer of lipids	[23]
	LDLR		rat	Pregnancy loss	fetal/offspring mortality	*LDLR* rat model for diabetes	[24]
**11**	***KCNK5***	Potassium Channel, Two Pore Domain Subfamily K, Member 5	human	Fertility - sperm volume	male potential fertility	*KCNK5* involved in protein and mRNA levels in sperm	[25]
	KCNK5		human	Male infertility	male potential fertility	*KCNK5* involvement in sperm inability to fertilize egg	[26]
	KCNK5		mouse	Male infertility	male potential fertility	*KCNK5* involved in sperm volume	[27]
	KCNK5		primate	Male fertility	male potential fertility	*KCNK5* involved in sperm function	[28]
	KCNK5		mouse	Female fertility	female potential fertility	*KCNK5* involved in oocyte survival/viability	[29]
	KCNK5		cattle	Lactation	reproductive	*KCNK5* expression	[30]

					outcomes		
**12**	***ABO***	ABO Blood Group (Transferase A, Alpha 1-3-N-Acetylgalactosaminyltransferase; Transferase B, Alpha 1-3-Galactosyltransferase)	human	Birth weight, maternal age at child-bearing	pregnancy outcomes	*ABO* variation effects	[31]
	ABO		human	Fetal growth restriction	pregnancy outcomes	*ABO* variation effects	[32]
	ABO		human	fetal hypoxia, pregnancy complications, hemolytic disease of fetus/newborn, fetal death	pregnancy outcomes	*ABO* incompatibility effects	[33]
	ABO		human	protection against malaria	pregnancy outcomes	*ABO* variation effects	[34]
	ABO		human	Age at menarche	reproductive outcomes	*ABO* blood group phenotypes	[35]
	ABO		human	male infertility	male potential fertility	*ABO* involved in sperm concentration/function	[36]
	ABO		human	pregnancy complications	pregnancy outcomes	*ABO* involved in preeclampsia	[37]
	ABO		human	female fertility, embryo implantation	female potential fertility	*ABO* variation effects	[38]
**13**	***SWAP70***	SWAP Switching B-Cell Complex 70kDa Subunit	monkey	female fertility, implantation, placentation	female potential fertility	*SWAP-70* expression effects	[39]
	SWAP70		human	fetal growth restriction	pregnancy outcomes	*SWAP70* involved in preeclampsia	[40]
**14**	***SH2B3***	SH2B Adaptor Protein 3	human	intrauterine/postnatal growth	pregnancy outcomes	*SH2B3* variation effects	[41]
	SH2B3		human	male testicular function	male potential fertility	*SH2B3* variation effects	[42]
**15**	***PEMT***	Phosphatidylethanolamine N-Methyltransferase	human	fetal growth, placental function	pregnancy outcomes	Choline metabolism/*PEMT* expression effects	[43]
	PEMT		human	premature birth	pregnancy outcomes	*PEMT* variation [744CC genotype] effects	[44]
	PEMT		human	sperm quality	male potential fertility	*PEMT* variation [27774G.C] effects	[45]
	PEMT		human	fetal growth, placental function	pregnancy outcomes	mRNA levels of *PEMT* involved in fetal/placental function	[46]
	PEMT		mouse	embryo survival/viability during pre-implantation	female potential fertility	*PEMT* expression effects	[47]
	PEMT		human	fetal development	pregnancy outcomes	*PEMT* expression effects	[48]
**16**	***MRAS***	Muscle RAS Oncogene Homolog	mouse	male testicular function	male potential fertility	*MRAS* expression effects	[49]
	MRAS			embryo implantation	female potential	*MRAS* regulation by androgen	[50]

					fertility	and progesterone receptors	
	MRAS		mouse	embryo pluripotency	female potential fertility	*MRAS* expression effects	[51]
	MRAS	Muscle RAS Oncogene Homolog	human	breastfeeding capacity	reproductive outcomes	*MRAS* expression effects	[52]
**17**	***KIAA1462***	*KIAA1462*	bird	offspring number	reproductive outcomes	*KIAA1462* expression effects	[53]
	KIAA1462		human	birth-related myometrial gene expression	pregnancy outcomes	*KIAA1462* expression effects	[54]
	KIAA1462		mouse	female reproduction	female potential fertility	*KIAA1462* highly expressed in oocytes & ovaries	[55]
	KIAA1462		pig	fetal growth	pregnancy outcomes	*KIAA1462* expression effects	[56]
	KIAA1462		human	embryo implantation	female potential fertility	*KIAA1462* significantly differentially expression	[57]
**18**	***GUCY1A3***	Guanylate Cyclase 1, Soluble, Alpha 3	cattle	embryo implantation	female potential fertility	*GUCY1A3* expression effects	[58]
	GUCY1A3			embryo implantation	female potential fertility	*GUCY1A3* expression effects	[59]
	GUCY1A3		human	placental functioning	pregnancy outcomes	*GUCY1A3* expression effects	[60]
	GUCY1A3		human	birth weight	pregnancy outcomes	*GUCY1A3* expression effects	[61]
	GUCY1A3		human	fetal growth, birthweight, postnatal growth & metabolism	pregnancy outcomes	*GUCY1A3* fetal genotype involved in fetal development	[62]
**19**	***CDKN2B-AS1***	CDKN2B Antisense RNA 1	human	fertility	female potential fertility	*CDKN2B-AS1* linked endometriosis	[63]
	CDKN2B-AS1		human	fetal growth restriction	pregnancy outcomes	*CDKN2B-AS1* variation	[64]
**20**	***ANKS1A***	Ankyrin Repeat And Sterile Alpha Motif Domain Containing 1A	cattle	fertility	female potential fertility	*ANKS1A* significant expression in endometrium and corpus luteum	[65]
	ANKS1A		cattle	fertility	female potential fertility	*ANKS1A* 6.7-fold significantly up-regulated in blastocysts	[66]
	ANKS1A		human/ mouse	male fertility	male potential fertility	*ANKS1A* expression	[67]
**21**	***PDGFD***	Platelet Derived Growth Factor D	human	female fertility	female potential fertility	*PDGFD* involved in ovarian hyperstimulation	[68]
	PDGFD		human	female reproduction	female potential fertility	*PDGFD* significantly expressed in oocytes	[69]
	PDGFD		mouse	male/female reproduction	female potential fertility	*PDGFD* significantly expression	[70]
	PDGFD			female reproductive function	female potential fertility	*PDGFD* significantly down-regulated in endometrium	[71]
	PDGFD		rat	female reproductive function	female potential fertility	*PDGFD* significantly expressed	[72]
	PDGFD		human	pregnancy complication, preeclampsia	pregnancy outcomes	*PDGFD* significantly down-regulated in placenta	[73]
**22**	***KSR2***	Kinase Suppressor Of Ras 2	mouse	male fertility	male potential fertility	*KSR2* -/- knockout mouse model for spermatogenesis	[74]
	KSR2		cattle	female reproductive	female potential	*KSR2* significantly up-	[75]

				function	fertility	regulated in epithelial cells	
	KSR2		mouse	offspring growth	fetal/offspring mortality	*KSR2* -/- knockout mouse model	[76]
**23**	***FLT1***	Fms-Related Tyrosine Kinase 1	human	fetal development	pregnancy outcomes	*FLT1* expression effects	[77]
	FLT1		mouse	offspring viability, fetal growth	pregnancy outcomes	*FLT1* knockdown effects	[78]
	FLT1		human	pregnancy loss	fetal/offspring mortality	FLT1 involved in immune responses to placental malaria	[79]
	FLT1			female reproduction	female potential fertility	*FLT1* significantly expression in oocytes	[80]
	FLT1		human	intrauterine growth restriction	pregnancy outcomes	*FLT1* significantly up-regulated	[81]
	FLT1		human	fetal growth	pregnancy outcomes	*FLT1* significantly expressed in placenta	[82]
	FLT1		human	female reproduction	female potential fertility	*FLT1* significantly expressed in oocytes	[83]
	FLT1		human	female reproduction, implantation	female potential fertility	*FLT1* significantly expressed in uterus	[84]
	FLT1		human	female reproduction	pregnancy outcomes	*FLT1* significantly expressed in placenta, fetal tissues	[85]
	FLT1		human	intrauterine growth restriction	pregnancy outcomes	*FLT1* significantly expressed during pregnancy	[86]
**24**	***ABCG5***	ATP-Binding Cassette, Sub-Family G, Member 5	rat	intrauterine growth restriction	pregnancy outcomes	rat model of IUGR	[87]
	ABCG5			trophoblast, blastocyst development	female potential fertility	*ABCG5* gene expression effects	[88]
**25**	***ZC3HC1***	Zinc Finger, C3HC-Type Containing 1		male fertility	male potential fertility	meiosis disruptors	[89]
	ZC3HC1		mouse	pregnancy establishment, maintenance, conceptus survival	female potential fertility	*ZC3HC1* expression, 1.57-fold significantly changed	[90]
**26**	***SMAD3***	SMAD Family Member 3		folliculogenesis	female potential fertility	*SMAD3* expression effects	[91]
	SMAD3		mouse/rat	oocyte function	female potential fertility	*SMAD3* expression effects	[92]
	SMAD3			estrogen receptor interactions	female potential fertility	*SMAD3* expression effects	[93]
	SMAD3		rat	testis function	male potential fertility	*SMAD3* expression effects	[94]
	SMAD3		human	age at natural menopause	reproductive outcomes	*SMAD3* interaction effects	[95]
	SMAD3		human	twinning capacity	reproductive outcomes	*SMAD3* genotype (rs17293443-C) effects	[96]
	SMAD3		human	female fertility and fecundity	female potential fertility	*SMAD3* promotes proliferation and steroidogenesis of human ovarian lutenized granulosa cells	[97]
	SMAD3		mouse	embryo viability	female potential fertility	*SMAD3* signalling effects	[98]

	SMAD3		human	spermatogenesis, male reproduction	male potential fertility	*SMAD3* expression effects	[99]
**27**	***SLC22A3***	Solute carrier family 22, extra neuronal monoamine transporter	human	placental functioning	pregnancy outcomes	*SLC22A3* expression effects	[100]
	SLC22A3		human	fetal development, fetal-placental resource provisioning	pregnancy outcomes	*SLC22A3* expression effects	[101]
	SLC22A3		human	fetal-placental functioning	pregnancy outcomes	*SLC22A3* expression changes during pregnancy	[102]
	SLC22A3		human	fetal-placental functioning	pregnancy outcomes	*SLC22A3* significantly expressed by trimester	[102]
**28**	***REST***	RE1-Silencing Transcription Factor	mouse	embryo functioning	female potential fertility	*REST* regulatory network effects	[103]
**29**	***PPAP2B***	Phospholipid Phosphatase 3	human	endometriosis, female fertility	female potential fertility	*PPAP2B* -1.69-fold significantly changed	[104]
	PPAP2B		human/rodent	gametogenesis	male potential fertility	*PPAP2B* expression effects	[105]
	PPAP2B		sheep	breeding capacity	reproductive outcomes	*PPAP2B* association effects	[106]
	PPAP2B		human	pregnancy complications	pregnancy outcomes	*PPAP2B* 1.36 -fold significantly up-regulated in placental tissues of preeclamptic mothers	[107]
	PPAP2B		human	embryo viability	female potential fertility	*PPAP2B* involved in spontaneous abortion due to parthenogenesis	[108]
	PPAP2B		human	embryo implantation	female potential fertility	*PPAP2B* differential expression effects	[57]
	PPAP2B			female reproductive function	female potential fertility	*PPAP2B* up-regulated in endometrium	[71]
**30**	***MIA3***	Melanoma Inhibitory Activity Family, Member 3	cattle	ovarian functioning	female potential fertility	*MIA3* 4.6-fold significantly up-regulated	[109]
	MIA3		mouse	placental (dys)function	female potential fertility	*MIA3* expresed in early trophoblast differentiation	[110]
**31**	***IL6R***	Interleukin 6 Receptor	pig	endometrium functioning	pregnancy outcomes	*IL6R* significantly differentially expressed in endometrium	[111]
	IL6R		cattle	endometrium functioning	pregnancy outcomes	*IL6R* 3.38-fold significantly up-regulated during pregnancy	[112]
	IL6R		human	endometrium functioning in PCOS women	female potential fertility	*IL6R* significantly up-regulated	[113]
	IL6R		human	pre-term birth SNP variation	pregnancy outcomes	*IL6R* significantly associated with pre-term birth	[114]
**32**	***HDAC9***	Histone Deacetylase 9	human	oocyte function	female potential fertility	*HDAC9* expression effects	[115]
	HDAC9		cattle	male fertility	male potential fertility	*HDAC9* involved in germ cell production	[116]
	HDAC9		pig	birth weight	pregnancy outcomes	*HDAC9* expression effects	[117]

	HDAC9		human/ mouse	oocyte function	female potential fertility	*HDAC9* expression effects	[118]
	HDAC9		human	birth-related myometrial gene expression	pregnancy outcomes	*HDAC9* expression effects	[119]
**33**	***COL4A1***	Collagen, Type IV, Alpha 1	pig	neonate survival	pregnancy outcomes	*COL4A1* expression effects	[120]
	COL4A1		human	testis function	male potential fertility	*COL4A1* expression effects	[121]
	COL4A1		mouse	folliculogenesis	female potential fertility	*COL4A1* expression effects	[122]
	COL4A1		human	fetal survival	fetal/offspring mortality	*COL4A1* mutation effects	[123]
	COL4A1			fetal/placenta growth and development	pregnancy outcomes	*COL4A1* expression effects	[124]
**34**	***ABHD2***	Abhydrolase Domain Containing 2		male fertility	male potential fertility	*ABHD2* expression effects	[125]
**35**	***SORT1***	Sortilin 1	human	endometrium functioning	pregnancy outcomes	*SORT1* significantly expressed during labour	[54]
	SORT1			ovarian functioning	female potential fertility	*SORT1* significantly up-regulated	[126]
	SORT1		rat	ovarian functioning	female potential fertility	*SORT1* expression effects	[127]
	SORT1		human	embryo implantation	female potential fertility	*SORT1* differential expression effects	[128]
**36**	***SLC22A5***	Solute Carrier Family 22 (Organic Cation/Carnitine Transporter), Member 5	mouse	male infertility	male potential fertility	*SLC22A5* mutation related to male infertility	[129]
	SLC22A5		pig	reproductive variation, offspring born alive and total born	reproductive outcomes	*SLC22A5* genotype effects on reproductive capacity	[130]
	SLC22A5		pig	age at puberty	reproductive outcomes	*SLC22A5* genotype effects	[131]
**37**	***NOA1***	Nitric Oxide Associated 1	human	male fertility, testicular functioning	male potential fertility	*NOA1* expression effects	[132]
	NOA1		mouse	embryo/trophoblast viability	female potential fertility	*NOA1*-deficient mouse model	[133]
**38**	***LPL***	Lipoprotein Lipase	human	pregnancy complications	pregnancy outcomes	*LPL* expression effects	[134]
	LPL		human	male infertility	male potential fertility	sperm DNA fragmentation related to *LPL* expression	[135]
	LPL		human	reproductive timing	reproductive outcomes	*LPL* expression effects	[7]
	LPL		human	intrauterine growth restriction	pregnancy outcomes	*LPL*-mediated fetal-placental nutrient transfer	[136]
	LPL		human/ mouse	placental functioning	pregnancy outcomes	*LPL* expression effects	[137]
	LPL			fetal/placental resource transfer, pregnancy	pregnancy outcomes	*LPL* expression effects	[138]

				complications			
	LPL		human	testis/spermatogene sis	male potential fertility	*LPL* expression effects	[139]
	LPL		mouse	placental regulation of cholesterol	pregnancy outcomes	*LPL* involved in maternal-fetal transfer of lipids	[23]
**39**	***COL4A2***	Collagen, Type IV, Alpha 2	mouse	fetal viability	fetal/offspring mortality	mouse knockout model for *COL4A2*	[140]
	COL4A2		human	testis function	male potential fertility	*COL4A2* expression effects	[121]
	COL4A2		human	offspring viability	fetal/offspring mortality	*COL4A2* expression effects	[141]
**40**	***ADAMST7***	ADAM Metallopeptidase With Thrombospondin Type 1 Motif, 7	mouse	embryogenesis	female potential fertility	*COL4A2* expression effects	[142]
	ADAMST7		dog	mammary tissue functioning	reproductive outcomes	*ADAMST7* significantly up-regulated in mammary tissues	[143]
	ADAMST7		human	breastfeeding capacity	reproductive outcomes	*ADAMST7* expression effects	[52]

Table footnotes:

***'fitness class' column defined further:**

male potential fertility - includes processes affecting spermatogenesis, sperm motility, volume or function that ultimately affect probability of successful egg fertilization.

female potential fertility - includes processes affecting embryogenesis (i.e. oocyte viability, survival), functioning of uterus (i.e. implantation receptivity, endometrium functioning), placentation (trophoblast cell motility) that ultimately affects initial successful establishment of pregnancy.

pregnancy outcomes - includes processes affecting regulation of blood pressure, nutrient and oxygen transfer between fetal and placental tissues during pregnancy that ultimately influences fetal growth, development and survival.

fetal/offspring mortality - includes processes linked to pregnancy defects, resistance to pathogens, affecting survival of fetus during pregnancy or perinatal mortality.

reproductive outcomes - includes effects on age at maturity, reproductive timing, potential number of offspring, breastfeeding capacity.

Search criteria:

- For each CAD gene, Google scholar was used to search for studies using the 'Search terms' (below) and the gene name (*BCAS3* is used as an example)
- For each search, only the first page of results was considered. Search results most consistent with all search terms are ranked by page, thus the most relevant results were always on the first page. This approach was also employed to keep this literature search tractable in terms of time (i.e. a search for each of the terms below for one gene usually took ~1 hour).
- We also used the GWAS Catalog (https://www.ebi.ac.uk/gwas/) using the gene name to search for further potential links to fitness related traits

Search terms (example using gene *BCAS3*):

- "*BCAS3*" and "reproduction" and gene and -"noncommercial use, distribution, and reproduction in any"
- "*BCAS3*" and "fitness" and gene
- "*BCAS3*" and "fertility" and gene
- "*BCAS3*" and "menarche" and gene
- "*BCAS3*" and "menopause" and gene
- "*BCAS3*" and "birth" or "birth weight"
- "*BCAS3*" and "pregnancy" and gene
- "*BCAS3*" and "placenta" and gene

- "*BCAS3*" and "implantation" and gene
- "*BCAS3*" and "oocyte" and gene
- "*BCAS3*" and "sperm" and gene
- "*BCAS3*" and "testis"

References

1. Siva, K., et al., *human BCAS3 expression in embryonic stem cells and vascular precursors suggests a role in human embryogenesis and tumor angiogenesis.* PLoS One, 2007. **2**(11): p. e1202.
2. Zuccotti, M., et al., *Maternal Oct-4 is a potential key regulator of the developmental competence of mouse oocytes.* BMC Developmental Biology, 2008. **8**(1): p. 1-14.
3. Nestler, A., et al., *Blood pressure in pregnancy and magnesium sensitive genes.* Pregnancy Hypertens, 2014. **4**(1): p. 41-5.
4. Gheorghe, C.P., et al., *Gene expression patterns in the hypoxic murine placenta: a role in epigenesis?* Reprod Sci, 2007. **14**(3): p. 223-33.
5. Yuan, H., et al., *A de novo triplication on 2q22.3 including the entire ZEB2 gene associated with global developmental delay, multiple congenital anomalies and behavioral abnormalities.* Mol Cytogenet, 2015. **8**: p. 99.
6. Bao, J.Q., et al., *UPF2, a nonsense-mediated mRNA decay factor, is required for prepubertal Sertoli cell development and male fertility by ensuring fidelity of the transcriptome.* Development, 2015. **142**(2): p. 352-362.
7. Spencer, K.L., et al., *Genetic Variation and Reproductive Timing: African American Women from the Population Architecture Using Genomics and Epidemiology (PAGE) Study.* Plos One, 2013. **8**(2).
8. Kakourou, G., et al., *Investigation of gene expression profiles before and after embryonic genome activation and assessment of functional pathways at the human metaphase II oocyte and blastocyst stage.* Fertility and Sterility, 2013. **99**(3): p. 803-+.
9. Saben, J., et al., *Early growth response protein-1 mediates lipotoxicity-associated placental inflammation: role in maternal obesity.* American Journal of Physiology-Endocrinology and Metabolism, 2013. **305**(1): p. E1-E14.
10. Zhou, L., et al., *Local injury to the endometrium in controlled ovarian hyperstimulation cycles improves implantation rates.* Fertility and Sterility, 2008. **89**(5): p. 1166-1176.
11. Newbold, R.R., et al., *Developmental exposure to diethylstilbestrol alters uterine gene expression that may be associated with uterine neoplasia later in life.* Molecular Carcinogenesis, 2007. **46**(9): p. 783-796.
12. Patel, O.V., et al., *Homeorhetic adaptation to lactation: comparative transcriptome analysis of mammary, liver, and adipose tissue during the transition from pregnancy to lactation in rats.* Functional & Integrative Genomics, 2011. **11**(1): p. 193-202.

13. Farkas, R.M., et al., *The Drosophila Cog5 homologue is required for cytokinesis, cell elongation, and assembly of specialized golgi architecture during spermatogenesis.* Molecular Biology of the Cell, 2003. **14**(1): p. 190-200.
14. Foulquier, F., *COG defects, birth and rise!* Biochimica Et Biophysica Acta-Molecular Basis of Disease, 2009. **1792**(9): p. 896-902.
15. Solca, C., G.S. Tint, and S.B. Patel, *Dietary xenosterols lead to infertility and loss of abdominal adipose tissue in sterolin-deficient mice.* Journal of Lipid Research, 2013. **54**(2): p. 397-409.
16. Dixon, P.H. and C. Williamson, *The pathophysiology of intrahepatic cholestasis of pregnancy.* Clinics and Research in Hepatology and Gastroenterology, 2016. **40**(2): p. 141-153.
17. Bi, W.M., et al., *Inactivation of Rai1 in mice recapitulates phenotypes observed in chromosome engineered mouse models for Smith-Magenis syndrome.* human Molecular Genetics, 2005. **14**(8): p. 983-995.
18. Girirajan, S. and S.H. Elsea, *Abnormal maternal behavior, altered sociability, and impaired serotonin metabolism in Rai1-transgenic mice.* Mammalian Genome, 2009. **20**(4): p. 247-255.
19. Shen, Z., et al., *Estradiol regulation of nucleotidases in female reproductive tract epithelial cells and fibroblasts.* PLoS One, 2013. **8**(7): p. e69854.
20. Horikoshi, M., et al., *New loci associated with birth weight identify genetic links between intrauterine growth and adult height and metabolism.* Nat Genet, 2013. **45**(1): p. 76-82.
21. Bhasin, K.K., et al., *Maternal low-protein diet or hypercholesterolemia reduces circulating essential amino acids and leads to intrauterine growth restriction.* Diabetes, 2009. **58**(3): p. 559-66.
22. Ethier-Chiasson, M., et al., *Influence of maternal lipid profile on placental protein expression of LDLr and SR-BI.* Biochem Biophys Res Commun, 2007. **359**(1): p. 8-14.
23. Overbergh, L., et al., *Expression of mouse alpha-macroglobulins, lipoprotein receptor-related protein, LDL receptor, apolipoprotein E, and lipoprotein lipase in pregnancy.* J Lipid Res, 1995. **36**(8): p. 1774-86.
24. McLean, M.P., Z. Zhao, and G.C. Ness, *Reduced hepatic LDL-receptor, 3-hydroxy-3-methylglutaryl coenzyme A reductase and sterol carrier protein-2 expression is associated with pregnancy loss in the diabetic rat.* Endocrine, 1995. **3**(10): p. 695-703.
25. Yeung, C.H. and T.G. Cooper, *Potassium channels involved in human sperm volume regulation--quantitative studies at the protein and mRNA levels.* Mol Reprod Dev, 2008. **75**(4): p. 659-68.
26. Cooper, T.G. and C.H. Yeung, *Involvement of potassium and chloride channels and other transporters in volume regulation by spermatozoa.* Curr Pharm Des, 2007. **13**(31): p. 3222-30.
27. Barfield, J.P., C.H. Yeung, and T.G. Cooper, *Characterization of potassium channels involved in volume regulation of human spermatozoa.* Mol Hum Reprod, 2005. **11**(12): p. 891-7.
28. Chow, G.E., et al., *Expression of two-pore domain potassium channels in nonhuman primate sperm.* Fertil Steril, 2007. **87**(2): p. 397-404.

29. Kang, D., et al., *TASK-2 Expression Levels are Increased in mouse Cryopreserved Ovaries.* Journal of Embryo Transfer, 2015. **30**(4): p. 277-282.
30. Wang, M., et al., *MicroRNA expression patterns in the bovine mammary gland are affected by stage of lactation.* J Dairy Sci, 2012. **95**(11): p. 6529-35.
31. Gloria-Bottini, F., et al., *Effect of smoking and ABO blood groups on maternal age at child bearing and on birth weight.* European Journal of Obstetrics & Gynecology and Reproductive Biology, 2011. **159**(1): p. 83-86.
32. Clark, P. and I.A. Greer, *The influence of maternal Lewis, Secretor and ABO(H) blood groups on fetal growth restriction.* J Thromb Haemost, 2011. **9**(12): p. 2411-5.
33. Dean, L., *Hemolytic disease of the newborn*, in *Hemolytic disease of the newborn*, L. Dean, Editor. 2005, National Center for Biotechnology Information (NCBI), National Library of Medicine, National Institutes of Health: Bethesda, MD.
34. Pathirana, S.L., et al., *ABO-blood-group types and protection against severe, Plasmodium falciparum malaria.* Ann Trop Med Parasitol, 2005. **99**(2): p. 119-24.
35. Balgir, R.S., *Menarcheal age in relation to ABO blood group phenotypes and haemoglobin-E genotypes.* J Assoc Physicians India, 1993. **41**(4): p. 210-1.
36. Omu, A.E., M. Al-Mutawa, and F. Al-Qattan, *ABO blood group and expression of antisperm antibodies in infertile couples in Kuwait.* Gynecol Obstet Invest, 1998. **45**(1): p. 49-53.
37. Franchini, M., C. Mengoli, and G. Lippi, *Relationship between ABO blood group and pregnancy complications: a systematic literature analysis.* Blood Transfusion, 2016. **5**: p. 1-8.
38. Mengoli, C., et al., *ABO blood group and fertility: a single-centre study.* Blood Transfus, 2015. **13**(3): p. 521-3.
39. Liu, J., et al., *Expression of SWAP-70 in the uterus and feto-maternal interface during embryonic implantation and pregnancy in the rhesus monkey (Macaca mulatta).* Histochem Cell Biol, 2006. **126**(6): p. 695-704.
40. Sitras, V., C. Fenton, and G. Acharya, *Gene expression profile in cardiovascular disease and preeclampsia: a meta-analysis of the transcriptome based on raw data from human studies deposited in Gene Expression Omnibus.* Placenta, 2015. **36**(2): p. 170-8.
41. Padidela, R., *Effects of polymorphisms in the growth hormone and insulin-like growth factor axis on intrauterine and postnatal growth*. 2013, University College London.
42. Slack, C., et al., *Regulation of lifespan, metabolism, and stress responses by the Drosophila SH2B protein, Lnk.* PLoS Genet, 2010. **6**(3): p. e1000881.
43. Hogeveen, M., et al., *Umbilical choline and related methylamines betaine and dimethylglycine in relation to birth weight.* Pediatr Res, 2013. **73**(6): p. 783-7.
44. Zhu, J., *Choline Intake During Pregnancy and Genetic Polymorphisms Influence Choline Metabolism in Chinese Preterms Receiving Total Parenteral Nutrition Therapy* The Faseb Journal, 2016. **30**.

45. Lazaros, L., et al., *Phosphatidylethanolamine N-methyltransferase and choline dehydrogenase gene polymorphisms are associated with human sperm concentration.* Asian J Androl, 2012. **14**(5): p. 778-83.
46. Khot, V., et al., *Expression of genes encoding enzymes involved in the one carbon cycle in rat placenta is determined by maternal micronutrients (folic acid, vitamin B12) and omega-3 fatty acids.* Biomed Res Int, 2014. **2014**: p. 613078.
47. Cheng, E.H., et al., *Requirement of Leukemia Inhibitory Factor or Epidermal Growth Factor for Pre-Implantation Embryogenesis via JAK/STAT3 Signaling Pathways.* PLoS One, 2016. **11**(4): p. e0153086.
48. Yan, J., et al., *Pregnancy alters choline dynamics: results of a randomized trial using stable isotope methodology in pregnant and nonpregnant women.* Am J Clin Nutr, 2013. **98**(6): p. 1459-67.
49. Gong, W., et al., *Transcriptome profiling of the developing postnatal mouse testis using next-generation sequencing.* Sci China Life Sci, 2013. **56**(1): p. 1-12.
50. Cloke, B., et al., *The androgen and progesterone receptors regulate distinct gene networks and cellular functions in decidualizing endometrium.* Endocrinology, 2008. **149**(9): p. 4462-74.
51. Palmqvist, L., et al., *Correlation of murine embryonic stem cell gene expression profiles with functional measures of pluripotency.* Stem Cells, 2005. **23**(5): p. 663-80.
52. Colodro-Conde, L., et al., *A twin study of breastfeeding with a preliminary genome-wide association scan.* Twin Res Hum Genet, 2015. **18**(1): p. 61-72.
53. Huang, C.-W., et al., *Efficient SNP Discovery by Combining Microarray and Lab-on-a-Chip Data for Animal Breeding and Selection.* Microarrays, 2015. **4**(4): p. 570-595.
54. Chan, Y.W., et al., *Assessment of myometrial transcriptome changes associated with spontaneous human labour by high-throughput RNA-seq.* Exp Physiol, 2014. **99**(3): p. 510-24.
55. Fledel-Alon, A., et al., *Variation in human recombination rates and its genetic determinants.* PLoS One, 2011. **6**(6): p. e20321.
56. Perez-Montarelo, D., et al., *Identification of genes regulating growth and fatness traits in pig through hypothalamic transcriptome analysis.* Physiological Genomics, 2014. **46**(6): p. 195-206.
57. Aghajanova, L., et al., *Comparative Transcriptome Analysis of human Trophectoderm and Embryonic Stem Cell-Derived Trophoblasts Reveal Key Participants in Early Implantation.* Biology of Reproduction, 2012. **86**(1).
58. Ponsuksili, S., et al., *Gene Expression and DNA-Methylation of Bovine Pretransfer Endometrium Depending on Its Receptivity after In Vitro-Produced Embryo Transfer.* Plos One, 2012. **7**(8).
59. Duzyj, C.M., et al., *PreImplantation factor (PIF*) promotes embryotrophic and neuroprotective decidual genes: effect negated by epidermal growth factor.* Journal of Neurodevelopmental Disorders, 2014. **6.**
60. Sedlmeier, E.M., et al., *human placental transcriptome shows sexually dimorphic gene expression and responsiveness to maternal dietary n-3 long-*

chain polyunsaturated fatty acid intervention during pregnancy. Bmc Genomics, 2014. **15**.
61. Taal, H.R., *Early Growth, Cardiovascular and Renal Development - The Generation R Study*. 2013, Erasmus University: Rotterdam.
62. Horikoshi, M., et al., *New loci associated with birth weight identify genetic links between intrauterine growth and adult height and metabolism.* Nature Genetics, 2013. **45**(1): p. 76-U115.
63. Pagliardini, L., et al., *Replication and meta-analysis of previous genome-wide association studies confirm vezatin as the locus with the strongest evidence for association with endometriosis.* human Reproduction, 2015. **30**(4): p. 987-993.
64. Sayed, A.-R.A.A.-R., *Molecular genetic studies in pregnancies affected by preeclampsia and intrauterine growth restriction*, in *School of Molecular Medical Sciences*. 2011, University of Nottingham: United Kingdom.
65. Moore, S.G., et al., *Differentially Expressed Genes in Endometrium and Corpus Luteum of Holstein Cows Selected for High and Low Fertility Are Enriched for Sequence Variants Associated with Fertility.* Biol Reprod, 2016. **94**(1): p. 19.
66. Gad, A.Y.M.H., *Transcriptome profiling of bovine blastocysts developed under alternative culture conditions during specific stages of development*. 2012, University of Bonn: Bonn, Germany.
67. Tuttelmann, F., et al., *Copy number variants in patients with severe oligozoospermia and Sertoli-cell-only syndrome.* PLoS One, 2011. **6**(4): p. e19426.
68. Scotti, L., et al., *Platelet-derived growth factor BB and DD and angiopoietin1 are altered in follicular fluid from polycystic ovary syndrome patients.* Mol Reprod Dev, 2014. **81**(8): p. 748-56.
69. Bonnet, A., et al., *Spatio-Temporal Gene Expression Profiling during In Vivo Early Ovarian Folliculogenesis: Integrated Transcriptomic Study and Molecular Signature of Early Follicular Growth.* PLoS One, 2015. **10**(11): p. e0141482.
70. Sharov, A.A., et al., *Effects of aging and calorie restriction on the global gene expression profiles of mouse testis and ovary.* BMC Biol, 2008. **6**: p. 24.
71. Popovici, R.M., et al., *Gene expression profiling of human endometrial-trophoblast interaction in a coculture model.* Endocrinology, 2006. **147**(12): p. 5662-5675.
72. Sleer, L.S. and C.C. Taylor, *Cell-type localization of platelet-derived growth factors and receptors in the postnatal rat ovary and follicle.* Biol Reprod, 2007. **76**(3): p. 379-90.
73. Sitras, V., et al., *Differential placental gene expression in severe preeclampsia.* Placenta, 2009. **30**(5): p. 424-33.
74. Moretti, E., et al., *Ultrastructural study of spermatogenesis in KSR2 deficient mice.* Transgenic Res, 2015. **24**(4): p. 741-51.
75. Cerny, K.L., et al., *A transcriptomal analysis of bovine oviductal epithelial cells collected during the follicular phase versus the luteal phase of the estrous cycle.* Reprod Biol Endocrinol, 2015. **13**: p. 84.
76. Henry, M.D., et al., *Obesity-dependent dysregulation of glucose homeostasis in kinase suppressor of ras 2-/- mice.* Physiol Rep, 2014. **2**(7).

77. Kaipainen, A., et al., *The Related Flt4, Flt1, and Kdr Receptor Tyrosine Kinases Show Distinct Expression Patterns in human Fetal Endothelial-Cells.* Journal of Experimental Medicine, 1993. **178**(6): p. 2077-2088.
78. Khankin, E.V., et al., *Hemodynamic, Vascular, and Reproductive Impact of FMS-Like Tyrosine Kinase 1 (FLT1) Blockade on the Uteroplacental Circulation During Normal mouse Pregnancy.* Biology of Reproduction, 2012. **86**(2).
79. Muehlenbachs, A., et al., *Natural selection of FLT1 alleles and their association with malaria resistance in utero.* Proceedings of the National Academy of Sciences of the United States of America, 2008. **105**(38): p. 14488-14491.
80. Bonnet, A., et al., *An overview of gene expression dynamics during early ovarian folliculogenesis: specificity of follicular compartments and bi-directional dialog.* Bmc Genomics, 2013. **14**.
81. Fritz, R., *Trophoblast Retrieval And Isolation From The Cervix (tric) For Non-Invasive Prenatal Genetic Diagnosis And Prediction Of Abnormal Pregnancy Outcome*, in *Physiology*. 2015, Wayne State University.
82. Korevaar, T.I., et al., *Soluble Flt1 and placental growth factor are novel determinants of newborn thyroid (dys)function: the generation R study.* J Clin Endocrinol Metab, 2014. **99**(9): p. E1627-34.
83. Dan, S., L. Haibo, and L. Hong, *Pathogenesis and stem cell therapy for premature ovarian failure.* OA Stem Cells, 2014. **10**(2).
84. Dey, S.K., et al., *Molecular cues to implantation.* Endocr Rev, 2004. **25**(3): p. 341-73.
85. Sood, R., et al., *Gene expression patterns in human placenta.* Proc Natl Acad Sci U S A, 2006. **103**(14): p. 5478-83.
86. Tsao, P.N., et al., *Excess soluble fms-like tyrosine kinase 1 and low platelet counts in premature neonates of preeclamptic mothers.* Pediatrics, 2005. **116**(2): p. 468-72.
87. Chen, L.H., et al., *[Effects of intrauterine growth restriction and high-fat diet on serum lipid and transcriptional levels of related hepatic genes in rats].* Zhongguo Dang Dai Er Ke Za Zhi, 2015. **17**(10): p. 1124-30.
88. Ozawa, M., et al., *Global gene expression of the inner cell mass and trophectoderm of the bovine blastocyst.* BMC Dev Biol, 2012. **12**: p. 33.
89. Archambeault, D.R. and M.M. Matzuk, *Disrupting the male germ line to find infertility and contraception targets.* Ann Endocrinol (Paris), 2014. **75**(2): p. 101-8.
90. Niklaus, A.L. and J.W. Pollard, *Mining the mouse transcriptome of receptive endometrium reveals distinct molecular signatures for the luminal and glandular epithelium.* Endocrinology, 2006. **147**(7): p. 3375-90.
91. Xu, J., J. Oakley, and E.A. McGee, *Stage-specific expression of Smad2 and Smad3 during folliculogenesis.* Biol Reprod, 2002. **66**(6): p. 1571-8.
92. Ethier, J.F. and J.K. Findlay, *Roles of activin and its signal transduction mechanisms in reproductive tissues.* Reproduction, 2001. **121**(5): p. 667-75.
93. Matsuda, T., et al., *Cross-talk between transforming growth factor-beta and estrogen receptor signaling through Smad3.* Journal of Biological Chemistry, 2001. **276**(46): p. 42908-42914.

94. Xu, R., et al., *Developmental and stage-specific expression of Smad2 and Smad3 in rat testis.* Journal of Andrology, 2003. **24**(2): p. 192-200.
95. Pyun, J.A., et al., *Genome-wide association studies and epistasis analyses of candidate genes related to age at menarche and age at natural menopause in a Korean population.* Menopause-the Journal of the North American Menopause Society, 2014. **21**(5): p. 522-529.
96. Mbarek, H., et al., *Identification of Common Genetic Variants Influencing Spontaneous Dizygotic Twinning and Female Fertility.* American Journal of human Genetics, 2016. **98**(5): p. 898-908.
97. Liu, Y.X., et al., *Effects of Smad3 on the Proliferation and Steroidogenesis in human Ovarian Luteinized Granulosa Cells.* Iubmb Life, 2014. **66**(6): p. 424-437.
98. Dunn, N.R., et al., *Combinatorial activities of Smad2 and Smad3 regulate mesoderm formation and patterning in the mouse embryo.* Development, 2004. **131**(8): p. 1717-1728.
99. Itman, C., et al., *Smad3 Dosage Determines Androgen Responsiveness and Sets the Pace of Postnatal Testis Development.* Endocrinology, 2011. **152**(5): p. 2076-2089.
100. Jacob, S. and K.H. Moley, *Gametes and embryo epigenetic reprogramming affect developmental outcome: Implication for assisted reproductive technologies.* Pediatric Research, 2005. **58**(3): p. 437-446.
101. Nelissen, E.C.M., et al., *Epigenetics and the placenta.* human Reproduction Update, 2011. **17**(3): p. 397-417.
102. Monk, D., et al., *Limited evolutionary conservation of imprinting in the human placenta.* Proceedings of the National Academy of Sciences of the United States of America, 2006. **103**(17): p. 6623-6628.
103. Johnson, R., et al., *REST Regulates Distinct Transcriptional Networks in Embryonic and Neural Stem Cells.* Plos Biology, 2008. **6**(10): p. 2205-2219.
104. Burney, R.O., et al., *Gene expression analysis of endometrium reveals progesterone resistance and candidate susceptibility genes in women with endometriosis.* Endocrinology, 2007. **148**(8): p. 3814-3826.
105. Chalmel, F., et al., *The conserved transcriptome in human and rodent male gametogenesis.* Proceedings of the National Academy of Sciences of the United States of America, 2007. **104**(20): p. 8346-8351.
106. Pokharel, K., et al., *Transcriptome profiling of Finnsheep ovaries during out-of-season breeding period.* Agricultural and Food Science, 2015. **24**(1): p. 1-9.
107. Yan, Y.H., et al., *Screening for preeclampsia pathogenesis related genes.* European Review for Medical and Pharmacological Sciences, 2013. **17**(22): p. 3083-3094.
108. Liu, N., et al., *Genome-wide Gene Expression Profiling Reveals Aberrant MAPK and Wnt Signaling Pathways Associated with Early Parthenogenesis.* Journal of Molecular Cell Biology, 2010. **2**(6): p. 333-344.
109. Hatzirodos, N., et al., *Transcriptome profiling of granulosa cells from bovine ovarian follicles during atresia.* Bmc Genomics, 2014. **15**.

110. Moore, A.C. and A. Sutherland, *Characterizing the Nature of the Xst199 Mutation and the Normal Function of the TANGO1 Protein in Trophoblast Cells.* Faseb Journal, 2011. **25**.
111. Ostrup, E., et al., *Differential Endometrial Gene Expression in Pregnant and Nonpregnant Sows.* Biology of Reproduction, 2010. **83**(2): p. 277-285.
112. Mansouri-Attia, N., et al., *Endometrium as an early sensor of in vitro embryo manipulation technologies.* Proceedings of the National Academy of Sciences of the United States of America, 2009. **106**(14): p. 5687-5692.
113. Yan, L., et al., *Expression of apoptosis-related genes in the endometrium of polycystic ovary syndrome patients during the window of implantation.* Gene, 2012. **506**(2): p. 350-354.
114. Anum, E.A., et al., *Genetic Contributions to Disparities in Preterm Birth.* Pediatric Research, 2009. **65**(1): p. 1-9.
115. Zhang, P., et al., *Distinct sets of developmentally regulated genes that are expressed by human oocytes and human embryonic stem cells.* Fertility and Sterility, 2007. **87**(3): p. 677-690.
116. Hering, D.M., K. Olenski, and S. Kaminski, *Genome-Wide Association Study for Sperm Concentration in Holstein-Friesian Bulls.* Reproduction in Domestic Animals, 2014. **49**(6): p. 1008-1014.
117. Zhang, L.F., et al., *Genome Wide Screening of Candidate Genes for Improving piglet Birth Weight Using High and Low Estimated Breeding Value Populations.* International Journal of Biological Sciences, 2014. **10**(3): p. 236-244.
118. Assou, S., et al., *The human cumulus-oocyte complex gene-expression profile.* human Reproduction, 2006. **21**(7): p. 1705-1719.
119. Chan, Y.W., et al., *Assessment of myometrial transcriptome changes associated with spontaneous human labour by high- throughput RNA- seq.* Experimental Physiology, 2014. **99**(3): p. 510-524.
120. Jiang, L., et al., *Expression of X-linked genes in deceased Neonates and surviving cloned female piglets.* Molecular Reproduction and Development, 2008. **75**(2): p. 265-273.
121. Chen, S.R., et al., *The Wilms Tumor Gene, Wt1, Maintains Testicular Cord Integrity by Regulating the Expression of Col4a1 and Col4a2.* Biology of Reproduction, 2013. **88**(3).
122. Borup, R., et al., *Competence Classification of Cumulus and Granulosa Cell Transcriptome in Embryos Matched by Morphology and Female Age.* Plos One, 2016. **11**(4).
123. Garel, C., et al., *Fetal intracerebral hemorrhage and COL4A1 mutation: promise and uncertainty.* Ultrasound in Obstetrics & Gynecology, 2013. **41**(2): p. 228-230.
124. Rodriguez-Zas, S.L., K. Schellander, and H.A. Lewin, *Biological interpretations of transcriptomic profiles in mammalian oocytes and embryos.* Reproduction, 2008. **135**(2): p. 129-139.
125. Gerhardt, K., *Progesterone and Endocannabinoid Interaction Alters Sperm Activation.* Biol Reprod, 2016. **95**(1): p. 9.

126. Zhao, Y., et al., *Effect of luteal-phase support on endometrial microRNA expression following controlled ovarian stimulation.* Reprod Biol Endocrinol, 2012. **10**: p. 72.
127. Wang, C.Q. and L.-S. Ll, *Effect of CdCl_2 on Expression of Sortilin(Sort1) in rat Ovary.* Reproduction and Contraception, 2012. **7**.
128. Aghajanova, L., et al., *Comparative transcriptome analysis of human trophectoderm and embryonic stem cell-derived trophoblasts reveal key participants in early implantation.* Biol Reprod, 2012. **86**(1): p. 1-21.
129. Toshimori, K., et al., *Dysfunctions of the epididymis as a result of primary carnitine deficiency in juvenile visceral steatosis mice.* Febs Letters, 1999. **446**(2-3): p. 323-326.
130. Mote, B.E., et al., *Identification of genetic markers for productive life in commercial sows.* Journal of Animal Science, 2009. **87**(7): p. 2187-2195.
131. Rempel, L.A., et al., *Association analyses of candidate single nucleotide polymorphisms on reproductive traits in swine.* Journal of Animal Science, 2010. **88**(1): p. 1-15.
132. Okada, H., et al., *Genome-wide expression of azoospermia testes demonstrates a specific profile and implicates ART3 in genetic susceptibility.* Plos Genetics, 2008. **4**(2).
133. Kolanczyk, M., et al., *NOA1 is an essential GTPase required for mitochondrial protein synthesis.* Molecular Biology of the Cell, 2011. **22**(1): p. 1-11.
134. Schmella, M.J., et al., *The-93T/G LPL Promoter Polymorphism Is Associated With Lower Third-Trimester Triglycerides in Pregnant African American Women.* Biological Research for Nursing, 2015. **17**(4): p. 429-437.
135. Intasqui, P., et al., *Sperm nuclear DNA fragmentation rate is associated with differential protein expression and enriched functions in human seminal plasma.* Bju International, 2013. **112**(6): p. 835-843.
136. Tabano, S., et al., *Placental LPL gene expression is increased in severe intrauterine growth-restricted pregnancies.* Pediatric Research, 2006. **59**(2): p. 250-253.
137. Lindegaard, M.L.S., et al., *Endothelial and lipoprotein lipases in human and mouse placenta.* Journal of Lipid Research, 2005. **46**(11): p. 2339-2346.
138. Lager, S. and T.L. Powell, *Regulation of nutrient transport across the placenta.* J Pregnancy, 2012. **2012**: p. 179827.
139. Nielsen, J.E., et al., *Lipoprotein lipase and endothelial lipase in human testis and in germ cell neoplasms.* Int J Androl, 2010. **33**(1): p. e207-15.
140. Kuo, D.S., C. Labelle-Dumais, and D.B. Gould, *COL4A1 and COL4A2 mutations and disease: insights into pathogenic mechanisms and potential therapeutic targets.* human Molecular Genetics, 2012. **21**: p. R97-R110.
141. Yoneda, Y., et al., *De novo and inherited mutations in COL4A2, encoding the type IV collagen alpha2 chain cause porencephaly.* Am J Hum Genet, 2012. **90**(1): p. 86-90.
142. Hurskainen, T.L., et al., *ADAM-TS5, ADAM-TS6, and ADAM-TS7, novel members of a new family of zinc metalloproteases. General features and genomic distribution of the ADAM-TS family.* J Biol Chem, 1999. **274**(36): p. 25555-63.

143. Rao, N.A., et al., *Gene expression profiles of progestin-induced canine mammary hyperplasia and spontaneous mammary tumors.* J Physiol Pharmacol, 2009. **60 Suppl 1**: p. 73-84.
