# Supplementary material for: Genetic loci associated with coronary artery disease harbor evidence of selection and antagonistic pleiotropy
Source: PLoS Genet. 2017 Jun 22;13(6):e1006328. doi: 10.1371/journal.pgen.1006328 (PMC5480811; doi:10.1371/journal.pgen.1006328)
Supplement: S5 Table — Counts are based on S1 Table, ‘fitness class’ column. Most fitness-related traits were related to female potential fertility (29 of 40 genes had these effects) and pregnancy outcomes (25 of 40 genes had these effects). Some genes had broad or specific effects on fitness-related traits. For example, number of fitness classes affected ranged from 6 for ABO (had fitness effects across all classes) to 1, for example CNNM2 (evidence for fitness effects in pregnancy outcomes class). (PDF) [file pgen.1006328.s008.pdf]

Table S5. Summary of types of pleiotropic connections between coronary artery disease (CAD) and fitness-related traits. Counts are based on Table S1, 'fitness class' column. Most fitness-related traits were related to female potential fertility (29 of 40 genes had these effects) and pregnancy outcomes (25 of 40 genes had these effects). Some genes had broad or specific effects on fitness-related traits. For example, number of fitness classes affected ranged from 6 for *ABO* (had fitness effects across all classes) to 1, for example *CNNM2* (evidence for fitness effects in pregnancy outcomes class).

no.	CAD Gene	male potential fertility	female potential fertility	sum(both male and female fertility)	pregnancy outcomes	reproductive outcomes	fetal/offspring mortality	sum (all columns)
**1**	***BCAS3***		1		1			**2**
**2**	***CNNM2***				1			**1**
**3**	***TEX41***				1			**1**
**4**	***SMG6***		1					**1**
**5**	***PHACTR1***		1		1	1		**3**
**6**	***COG5***	1			1			**2**
**7**	***ABCG8***	1	1	1	1		1	**5**
**8**	***RAI1***				1			**1**
**9**	***NT5C2***		1		1			**2**
**10**	***LDLR***				1		1	**2**
**11**	***KCNK5***	1	1	1		1		**4**
**12**	***ABO***	1	1	1	1	1	1	**6**
**13**	***SWAP70***		1		1			**2**
**14**	***SH2B3***	1			1		1	**3**
**15**	***PEMT***	1	1	1	1			**4**
**16**	***MRAS***	1	1	1		1		**4**
**17**	***KIAA1462***		1		1	1		**3**
**18**	***GUCY1A3***		1		1		1	**3**
**19**	***CDKN2B-AS1***		1		1			**2**
**20**	***ANKS1A***	1	1	1				**3**
**21**	***PDGFD***		1		1			**2**
**22**	***KSR2***	1	1	1			1	**4**
**23**	***FLT1***		1		1		1	**3**
**24**	***ABCG5***		1		1			**2**
**25**	***ZC3HC1***	1	1	1				**3**
**26**	***SMAD3***	1	1	1		1		**4**
**27**	***SLC22A3***				1			**1**

**28**	***REST***		1					**1**
**29**	***PPAP2B***	1	1	1	1	1		**5**
**30**	***MIA3***		1					**1**
**31**	***IL6R***		1		1			**2**
**32**	***HDAC9***	1	1	1	1			**4**
**33**	***COL4A1***	1	1	1	1		1	**5**
**34**	***ABHD2***	1						**1**
**35**	***SORT1***		1		1			**2**
**36**	***SLC22A5***	1				1		**2**
**37**	***NOA1***	1	1	1				**3**
**38**	***LPL***	1			1	1		**3**
**39**	***COL4A2***	1					1	**2**
**40**	***ADAMST7***		1			1		**2**
	**sum**	**19**	**29**	**13**	**25**	**10**	**9**	**106**
							**average**	**2.7**
