# Supplementary material for: Genetic loci associated with coronary artery disease harbor evidence of selection and antagonistic pleiotropy
Source: PLoS Genet. 2017 Jun 22;13(6):e1006328. doi: 10.1371/journal.pgen.1006328 (PMC5480811; doi:10.1371/journal.pgen.1006328)
Supplement: S6 Table — Fitness-related traits include fertility potential, reproductive outcomes, pregnancy outcomes, fetal growth and survival, i.e. affecting the ability of an organism to reproduce and transfer genes to the next generation. The first column gives coronary artery disease (CAD) gene (first 20 of 40 CAD genes from Fig 1B/S1 Table). Columns 2–3 give name (abbreviated, full) of randomly chosen genes matched for approximate length for each CAD gene. Columns 4–8 provide key details of each study where random genes also contribute to traits that influence fitness, including what species that was demonstrated in, what biological process or fitness effects that gene is impacting, what fitness class that effect is likely to impact (e.g. dysfunctional spermatogenesis or embryogenesis will affect male and female fertility, ability to conceive), what the observed genetic effect or mechanism that gene was associated with. (PDF) [file pgen.1006328.s009.pdf]

Table S6. Pleiotropic links between randomly chosen genes and early-life fitness-related traits. Fitness-related traits include fertility potential, reproductive outcomes, pregnancy outcomes, fetal growth and survival, i.e. affecting the ability of an organism to reproduce and transfer genes to the next generation. The first column gives coronary artery disease (CAD) gene (first 20 of 40 CAD genes from Fig. 1B/Table S1). Columns 2-3 give name (abbreviated, full) of randomly chosen genes matched for approximate length for each CAD gene. Columns 4-8 provide key details of each study where random genes also contribute to traits that influence fitness, including what species that was demonstrated in, what biological process or fitness effects that gene is impacting, what fitness class that effect is likely to impact (e.g. dysfunctional spermatogenesis or embryogenesis will affect male and female fertility, ability to conceive), what the observed genetic effect or mechanism that gene was associated with.

CAD gene	Random Gene	full name	species	fitness effects	fitness class*	observed genetic effect or mechanism	ref
BCAS3	***STPG2***	Sperm Tail PG-Rich Repeat Containing 2	-	-	-	-	
CNNM2	***CFAP44***	Cilia And Flagella Associated Protein 44	-	-	-	-	
TEX41	***SHISA9***	Shisa Family Member 9	-	-	-	-	
SMG6	***TANGO6***	Transport And Golgi Organization 6 Homolog	-	-	-	-	
PHACTR1	***SUMF1***	Sulfatase Modifying Factor 1	mouse	embryogenesis	female potential fertility	*SUMF1* significantly up-regulated in developmentally incompetent mouse oocytes	[1]
COG5	***FRMD5***	FERM Domain Containing 5	-	-	-	-	
ABCG8	***ASIC5***	Acid Sensing Ion Channel Subunit Family Member 5	-	-	-	-	
RAI1	***ZNF516***	Zinc Finger Protein 516	human	endometriosis	female potential fertility	*ZNF516* appears to be involved in endometriosis	[2]
NT5C2	***LANCL1-AS1***	LANCL1 Antisense RNA 1	-	-	-	-	
LDLR	***SYT13***	Synaptotagmin 13	-	-	-	-	
KCNK5	***FAM53A***	Family With Sequence Similarity 53 Member A	-	-	-	-	
ABO	***TTC22***	Tetratricopeptide Repeat Domain 22	-	-	-	-	
SWAP70	***RNF157***	Ring Finger Protein 157	cattle	oocyte/follicle maturation (oocyte quality)	female potential fertility	In cattle model, *RNF157* 2.24 significantly differentially up-regulated between BCB+ and BCB- oocytes	[3]
	RNF157		human	early peripheral blood gene	pregnancy complications/ou	*RNF157* is -1.65 fold significantly (P=0.01)	[4]

				expression during pregnancy related to preeclampsia	tcomes	down-regulated in peripheral blood
SH2B3	***PLBD1-AS1***	PLBD1 Antisense RNA 1	-	-	-	-
PEMT	***WAC***	WW Domain Containing Adaptor With Coiled-Coil	-	-	-	-
MRAS	***TMEM178A***	Transmembrane Protein 178A	-	-	-	-
KIAA1462	***PLEKHD1***	Pleckstrin Homology And Coiled-Coil Domain Containing D1	-	-	-	-
GUCY1A3	***MACC1***	Metastasis Associated In Colon Cancer 1	-	-	-	-
CDKN2B-AS1	***CACNA2D4***	Calcium Voltage-Gated Channel Auxiliary Subunit Alpha2delta 4	-	-	-	-
ANKS1A	***NWD2***	NACHT And WD Repeat Domain Containing 2	-	-	-	-

Table footnotes:

***'fitness class' column defined further:**

male potential fertility - includes processes affecting spermatogenesis, sperm motility, volume or function that ultimately affect probability of successful egg fertilization.

female potential fertility - includes processes affecting embryogenesis (i.e. oocyte viability, survival), functioning of uterus (i.e. implantation receptivity, endometrium functioning), placentation (trophoblast cell motility) that ultimately affects initial successful establishment of pregnancy.

pregnancy outcomes - includes processes affecting regulation of blood pressure, nutrient and oxygen transfer between fetal and placental tissues during pregnancy that ultimately influences fetal growth, development and survival.

fetal/offspring mortality - includes processes linked to pregnancy defects, resistance to pathogens, affecting survival of fetus during pregnancy or perinatal mortality.

reproductive outcomes - includes effects on age at maturity, reproductive timing, potential number of offspring, breastfeeding capacity.

Search criteria:

- For each random gene, Google scholar was used to search for studies using the 'Search terms' (below) and the gene name (*STPG2* is used as an example)
- For each search, only the first page of results was considered. Search results most consistent with all search terms are ranked by page, thus the most relevant results were always on the first page. This approach was also employed to keep this literature search tractable in terms of time (i.e. a search for each of the terms below for one gene usually took ~1 hour).
- We also used the GWAS Catalog (https://www.ebi.ac.uk/gwas/) using the gene name to search for further potential links to fitness related traits

Search terms (example using gene *STPG2*):

- "*STPG2*" and "reproduction" and gene and -"noncommercial use, distribution, and reproduction in any"
- "*STPG2*" and "fitness" and gene
- "*STPG2*" and "fertility" and gene
- "*STPG2*" and "menarche" and gene

- "*STPG2*" and "menopause" and gene
- "*STPG2*" and "birth" or "birth weight"
- "*STPG2*" and "pregnancy" and gene
- "*STPG2*" and "placenta" and gene
- "*STPG2*" and "implantation" and gene
- "*STPG2*" and "oocyte" and gene
- "*STPG2*" and "sperm" and gene
- "*STPG2*" and "testis"

References

1. Zuccotti, M., et al., *Maternal Oct-4 is a potential key regulator of the developmental competence of mouse oocytes.* BMC Developmental Biology, 2008. **8**(1): p. 1-14.
2. Sun, P.R., et al., *Genome-wide profiling of long noncoding ribonucleic acid expression patterns in ovarian endometriosis by microarray.* Fertil Steril, 2014. **101**(4): p. 1038-46 e7.
3. Janowski, D., et al., *Incidence of apoptosis and transcript abundance in bovine follicular cells is associated with the quality of the enclosed oocyte.* Theriogenology, 2012. **78**(3): p. 656-659.
4. Enquobahrie, D.A., et al., *Maternal peripheral blood gene expression in early pregnancy and preeclampsia.* Int J Mol Epidemiol Genet, 2011. **2**(1): p. 78-94.
