# Supplementary material for: Genetic loci associated with coronary artery disease harbor evidence of selection and antagonistic pleiotropy
Source: PLoS Genet. 2017 Jun 22;13(6):e1006328. doi: 10.1371/journal.pgen.1006328 (PMC5480811; doi:10.1371/journal.pgen.1006328)
Supplement: S1 Discussion — Extended discussion on candidate adaptive signals found on coronary artery disease (CAD) loci in relation to the polygenic model of selection and previous studies examining genomic selection on broader cardiovascular disease loci. (PDF) [file pgen.1006328.s010.pdf]

Widespread signals of positive selection on CAD loci

Evidence of candidate positive selection signals for CAD loci were widespread, with many genes having significant iHS scores of small-medium size (i.e. iHS score range: 2-3) with four genes (*BCAS3*, *ANKS1A*, *CXCL12*, *PEMT*) harboring large selection signals (iHS >4), two of which had previously been identified as having strong selection signals including *BCAS3* (Breast Carcinoma Amplified Sequence 3) in the HapMap3 CEU population [1] and *PHACTR1* (phosphatase and actin regulator 1) across the ASW, CEU and CHB/CHD HapMap3 populations [2]. Twelve genes contained SNPs with selection scores that remained significant after correction for multiple testing (Fig. 1A). The consistency of smaller, less significant selection signals for several genes within most populations (i.e. *CNNM2*, *PHACTR1*, *PDGFD*) strongly suggest that these may be smaller and possibly valid incomplete selective sweeps that are typically missed due to stringency of multiple-correction thresholds and lack of validation across multiple populations.

These patterns match expectations from the polygenic model of selection that predicts that selection on complex traits mostly involves smaller shifts in many underlying loci; it is the likely reason why so few large selection signals have been found underlying complex traits in general [3, 4] and those underlying cardiovascular disease phenotypes in particular [5, 6]. For example, Kullo & Ding 2007 [6] found that 110 out of 364 genes in pathways associated with cardiovascular disease (i.e. inflammation, insulin, p53, Ras, cholesterol biosynthesis etc) had significantly higher Fst (empirical $P<0.05$) in at least one SNP between 4 populations, but none remained significant after correction for multiple testing. In a later study, Ding & Kullo 2011 [5] found that 8 out of 158 genome-wide significant SNPs in genes for 36 cardiovascular disease phenotypes and related traits (CHD, hypertension, stroke, BMI, lipids etc) had significantly elevated Fst between 52 populations in the Human Genome Diversity Project.

It is difficult to compare selection candidates we found in the 76 CAD associated genes with results from these two previous studies as full sets of gene lists and Fst estimates were not available for either, and they used loci underlying much broader cardiovascular disease phenotypes than our more current list of specific CAD loci [7]. Nevertheless, due to fine-scale imputation with the 1000 Genomes Panel, our study suggests that many more loci related to cardiovascular disease have been recently modified by natural selection than previously identified. The larger sample of SNPs also likely improved reliability of iHS p values, with many more estimates available per MAF bin used to standardize iHS measures [8].

The Fst measures used in the Ding and Kullo studies also differ qualitatively from the iHS scores we used. Fst captures allele frequency differences between populations and is less sensitive to detecting alleles that have undergone recent selection [9], while the iHS statistic detects whether common alleles are carried on unusually long haplotypes within populations and should be better at capturing more recent smaller selection signals [8]. Lastly, by considering not just genome-wide significant index SNPs, we were able to detect smaller selection signals within CAD loci that were consistent across populations and would have otherwise been missed. *PHACTR1* is a good example of this – several smaller candidate selection signals were found (iHS ranging from 2-3.8) where peak selection signals did not span the index SNP location - sometimes signals were in different introns within the same locus (Fig. S2).

References

1. Sabeti, P.C., et al., *Genome-wide detection and characterization of positive selection in human populations.* Nature, 2007. **449**(7164): p. 913-U12.
2. Williamson, S.H., et al., *Localizing recent adaptive evolution in the human genome.* Plos Genetics, 2007. **3**(6): p. 901-915.
3. Fu, W. and J.M. Akey, *Selection and adaptation in the human genome.* Annu Rev Genomics Hum Genet, 2013. **14**: p. 467-89.
4. Hernandez, R.D., et al., *Classic Selective Sweeps Were Rare in Recent Human Evolution.* Science, 2011. **331**(6019): p. 920-924.
5. Ding, K.Y. and I.J. Kullo, *Geographic differences in allele frequencies of susceptibility SNPs for cardiovascular disease.* Bmc Medical Genetics, 2011. **12**.
6. Kullo, I.J. and K.Y. Ding, *Patterns of population differentiation of candidate genes for cardiovascular disease.* Bmc Genetics, 2007. **8**.
7. Nikpay, M., et al., *A comprehensive 1000 Genomes-based genome-wide association meta-analysis of coronary artery disease.* Nature Genetics, 2015. **47**(10): p. 1121-+.
8. Voight, B.F., et al., *A map of recent positive selection in the human genome.* PLoS Biol, 2006. **4**(3): p. e72.
9. Sabeti, P.C., et al., *Positive natural selection in the human lineage.* Science, 2006. **312**(5780): p. 1614-20.
